# Graphene-based optofluidic tweezers for refractive-index and size-based nanoparticle sorting, manipulation, and detection

**DOI:** 10.1038/s41598-023-29122-w

**Published:** 2023-02-03

**Authors:** Elnaz Gholizadeh, Behnam Jafari, Saeed Golmohammadi

**Affiliations:** Faculty of Electrical and Computer Engineering, University of Tabriz, Tabriz, 5166616471 Iran

**Keywords:** Applied optics, Optical materials and structures, Optical physics, Optical techniques, Other photonics

## Abstract

This work proposes a novel design composed of graphene nanoribbons-based optofluidic tweezers to manipulate and sort bio-particles with radii below 2.5 nm. The suggested structure has been numerically investigated by the finite difference time domain (FDTD) method employing Maxwell's stress tensor analysis (MST). The finite element method (FEM) has been used to obtain the electrostatic response of the proposed structure. The tweezer main path is a primary channel in the center of the structure, where the microfluidic flow translates the nanoparticle toward this channel. Concerning the microfluid's drag force, the nanoparticles tend to move along the length of the main channel. The graphene nanoribbons are fixed near the main channel at different distances to exert optical forces on the moving nanoparticles in the perpendicular direction. In this regard, sub-channels embedding in the hBN layer on the Si substrate deviate bio-particles from the main path for particular nanoparticle sizes and indices. Intense hotspots with electric field enhancements up to 900 times larger than the incident light are realized inside and around the graphene ribbons. Adjusting the gap distance between the graphene nanoribbon and the main channel allows us to separate the individual particle with a specific size from others, thus guiding that in the desired sub-channel. Furthermore, we demonstrated that in a structure with a large gap between channels, particles experience weak field intensity, leading to a low optical force that is insufficient to detect, trap, and manipulate nanoparticles. By varying the chemical potential of graphene associated with the electric field intensity variations in the graphene ribbons, we realized tunability in sorting nanoparticles while structural parameters remained constant. In fact, by adjusting the graphene Fermi level via the applied gate voltage, nanoparticles with any desired radius will be quickly sorted. Moreover, we exhibited that the proposed structure could sort nanoparticles based on their refractive indices. Therefore, the given optofluidic tweezer can easily detect bio-particles, such as cancer cells and viruses of tiny size.

## Introduction

The development of microfluidic and optofluidic systems is going to trigger a revolution in different fields such as physics, biology, chemistry, medicine, and photonics. The unique characteristics of such fluidic systems include fast and non-destructive performance, low cost, high efficiency, multiple applications, and compact footprint. Also, microfluidic cell sorting systems have received much attention with a variety of methods for active control of cell movements or flow, such as electrokinetic mobilization of fluids for bacterial cell sorting^1,2^ and dielectrophoretic forces^3^. Nevertheless, the vulnerability of cells under highly intense fields, low speed, and buffer incompatibilities impairs the efficiency of conventional microfluidic designs. Another technique to manipulate and sort cells in hydrodynamic flow control is based on either on-chip or off-chip, which is utilized to sort living cells due to less vulnerability of cells under a high electric field. However, this method suffers from the slow cycle time of the mechanical switch and the relatively large volume of fluids in each cycle^4,5^.

In this line of research, optical tweezers for cell trapping and manipulation were first introduced by Ashkin et al. in 1987^6^. The radiation pressure of a focused laser beam resulting from the light momentum variations has been investigated to trap or push a single cell or particle in a fluidic medium without any physical contact. The imposed force on a particle depends on the size and optical properties of the particle as well as the surrounding fluidic medium. This optically-induced method opened a new promising approach to cell sorting networks in a microfluidic medium. The first single-cell sorting system was introduced at^7^, allowing the single cells to be trapped or sorted by imposed optical forces. Hence, this technique solved the problems mentioned regarding its non-invasive nature and ability to operate with a single cell.

The operation principle of conventional optical tweezers in the literature^8^ is based on optical far-field interactions. In these tweezers, the focal spot of a big numerical aperture (NA) objective has been used along prolonged paths to be more effective at far distances. However, these prolonged trap paths lead to drawbacks. First, observing the dynamic response of single molecules is impossible, and second, the diffraction limit restricts the sub-wavelength design of these tweezers. Therefore, an optical tweezer based on a near field or evanescent wave was developed to solve this limitation^9^. In the context of optical tweezers, it has been demonstrated that the optical force of an evanescent field is adequate to manipulate and trap microparticles at the interface of two media with different refractive indices, such as glass/water interfaces.

Nevertheless, such evanescent waves are not strong enough to trap and manipulate nanoparticles. Therefore, a metallic tip or nanoaperture has been employed for exciting surface plasmons waves (SP) to improve near-field enhancement. However, despite their ability to displace nanometer-sized single cells, the induced surface plasmons in a metallic tip introduce huge absorption losses reducing trapping stability^10,11^.

In recent decades, graphene plasmonic-based applications have been attracted significant attention such as sensors^12–16^, modulator^17,18^ photodetectors^19–23^. Especially in the recent years graphene plasmonics applications in optical tweezers (PT)^25–31^, have been introduced to address mentioned drawbacks and have been utilized in optofluidic systems to sort nanoparticles. For instance, in^25^, active graphene plasmonic tweezers and size/RI-based nanoparticle trapping and sorting with radii in the range of 5–50 nm is proposed. The structure is illuminated by a laser beam in the mid-IR frequency range of 4–8 µm and its wavelength and intensity are kept constant even for sorting functionality. Each unit cell of the structure is composed of a graphene layer on top of a metallic nanoring which is embedded in SiO_2_. Strong hotspots with field enhancements reaching values as high as 150 are obtained inside the rings with no need for sharp edges conventional in plasmonic structures. Graphene, as a two-dimensional material, with superior optoelectronic characteristics such as broad-band light absorption, high electrical and thermal conductivity, ultrafast charge carrier dynamics, and gate tunable charge carrier density is a promising alternative to metallic counterparts for designing plasmonic structures. However, the unique features of graphene are vulnerable to its substrate, originating from the interaction of each graphene atom with the surrounding medium^32^.

To improve graphene performance, different substrates, such as Co^33^, Ni^34^, Ru^35^, Pt^36^, SiC^37,38^, and SiO_2_ for graphene have been studied so far. Reports demonstrate that SiO_2_ as a conventional and widely used graphene substrate deals with problems such as carrier mobility reduction via scattering in the charged surface states, impurities, substrate surface roughness, and surface optical phonons. Therefore, a substrate is required for graphene-based structures without limiting its unique properties. Meanwhile, studies show that (hexagonal boron nitride) hBN with promising features as the graphene substrate^32,39,40^ can surpass other materials.

Dean et al. demonstrated the first graphene-based transistors using the hBN substrate with significant improvements^40^. This improvement originated from graphene mobility due to the higher reduction of surface impurities associated with the low lattice mismatch between the graphene and its hBN substrate. The graphene/hBN lattice mismatch is only 1.7% which is ten times smaller than graphene's conventional substrates like SiO_2_^40^. The flat surface of hBN, no dangling bonds, and no trap charges on the hBN surface make it a perfect substrate for a 2D graphene sheet^41^. High carrier mobility improves the plasmonic wave propagation and the optical response of graphene^32,41,42^. In addition, hBN has excellent physical properties such as high-temperature stability, corrosion resistance, sizeable optical absorption^43^, neutron capture interface, ultra-long carrier lifetime^39^, and significant negative electron affinity^32,40,44^. The unique feature of hBN/ graphene sandwich is investigated thoroughly in the graphene plasmonics Fabry–Perot wave interferometer photodetector^19^ in which by using hBN as an substrate for graphene in compare with the conventional substrates such as SiO_2_^45^. In this work we represented that using hBN instead of SiO_2_ reaches the responsivity of photodetector more than 10 times larger, more over due to high graphene mobility on hBN, the photodetector bandwidth experienced an immense improvement as well as photodetection speed. However, using hBN due to 2D nature of this material causes some irregularities on graphene features which is completely studied at our previous work^46^.

This study proposes a novel structure composed of graphene nanoribbon embedded in hBN to trap nanoparticles with radii in the 2.5–50 nm range and sort below R = 2.5 nm nanoparticles. The principal mechanism of nanoparticle manipulation relies on exciting graphene plasmon with a parallel-polarized incident light concerning the graphene surface. An extra optical force is exerted on nanoparticles via graphene nanoribbons perpendicular to the liquid drag force. The moving nanoparticles inside the liquid deviate from their original path in the main channel in response to exerted optical force in the vertical direction (F_x_), thus directing toward the adjacent nanoribbons where the electric field is significantly confined. The electric field’s hot spots on the graphene nanoribbons are 900 times larger than the incident one. The electric field enhancement on the graphene nanoribbons is significantly sensitive to graphene chemical potential. Therefore, the optical forces in the vertical (x) direction on nanoparticles can be tuned by correctly choosing the gate bias and the optimized gap distance between the main channel and graphene nanoribbons. Thus, a desired deviation of the nanoparticle allows us to direct it toward the outlet branches. The resonance wavelength of graphene ribbons is adjusted by tuning the graphene Fermi level, allowing us to determine the operating wavelength interval of the proposed optofluidic system. Finally, repetition of this process leads to individual particles with desired sizes or refractive indices being sorted and directed to outlet branches.

## Structure and method

**Figure 1:**
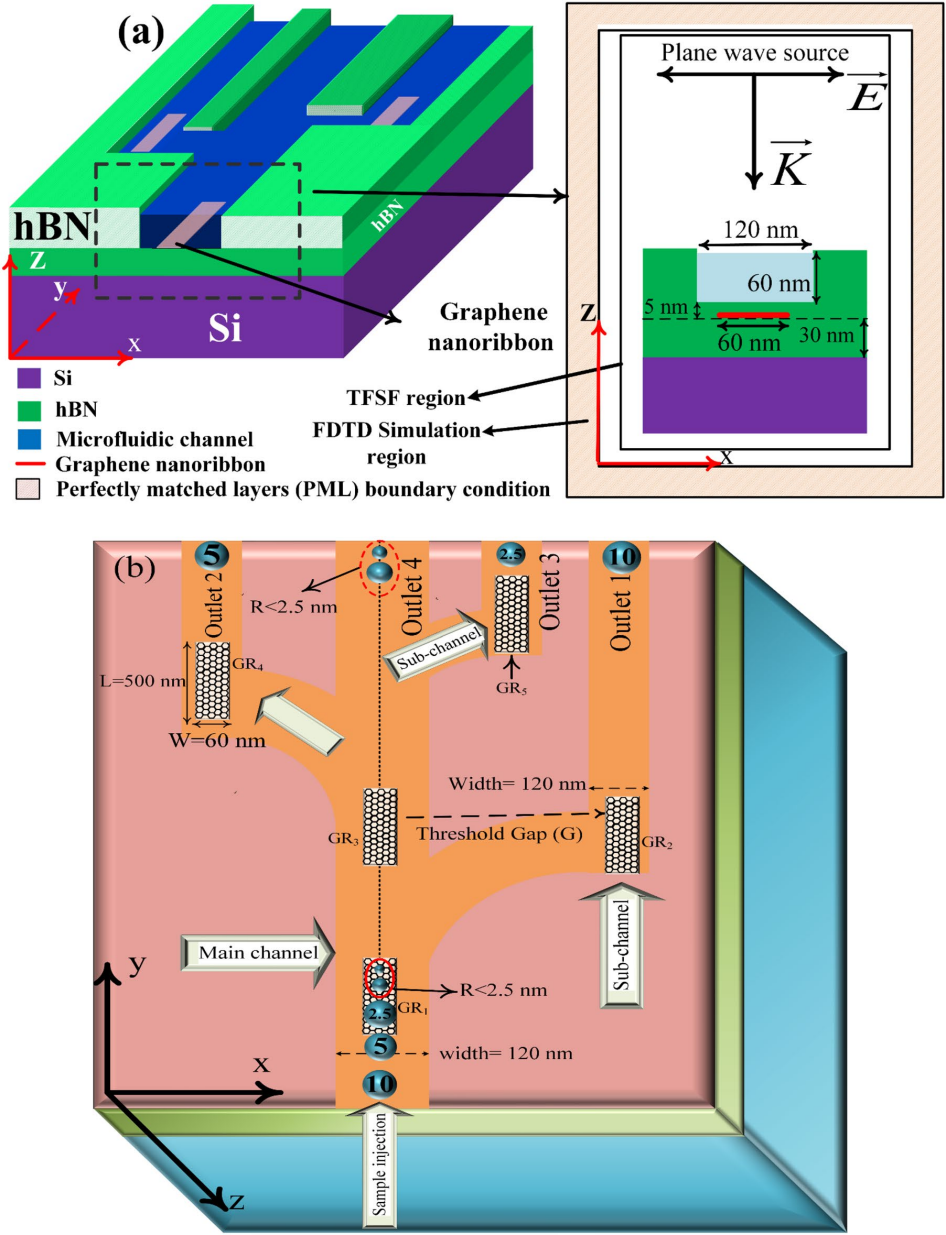
(**a**) 3-D schematic of the optofluidic nanoparticle sorter, (**b**) top view of the proposed Tweezer.

Figure 1 indicates the 3D schematic of the proposed structure. The graphene ribbons with 60 nm width and 500 nm length are embedded in the hBN layer. The hBN layers are fixed at the graphene nanoribbons' top and bottom. hBN as a spacer layer isolates the graphene ribbon from Si, acting as the graphene gate at the bottom, and also avoids the collapse of nanoparticles at the structure's top inside the microfluidic channel. The thickness of the top and bottom hBN layers is 30 nm, however, its thickness reduces to 5 nm inside the microfluidic channels as seen in the cross-section schematic in Fig. 1a. The target nanoparticles are assumed to be polystyrene spheres with *n* = 1.57 for radii in the range of 2.5 nm to 50 nm. The fluidic medium is considered to be water with *n* = 1.33. The electromagnetic wave simulation using the Maxwell Stress Tensor (MST) method is performed to calculate the imposed optical forces numerically. The primary channel is where the nanoparticles with a liquid are pushed in, as seen in Fig. 1b. The sorting mechanism starts with the largest size particle. For instance, the paths of diverse nanoparticles with different radii from 10 nm to below 2.5 nm have been represented. It is illustrated that the exerted force experienced by the large particles is larger than the smaller ones. Therefore, the first nanoribbon is fixed on the first sub-channel with a larger gap size than other gaps due to the immense force sensed by 10 nm particles. Similarly, the gap for sorting 5 nm particles is smaller than the gap of 10 nm size particles and larger than that of 2.5 nm size particles. The top view of the proposed structure is shown in Fig. 1b.

From Fig. 1b, it can be seen the sorting algorithm of nanoparticles step by step. In step 1, all particles enter from the center of the main channel in the y-direction, after passing through the first graphene nanoribbon (GR_1_), all the particles are placed in the middle of the ribbon as indicated by the dashed line in Fig. 1b, as a result of the vertical force of GR_1_ in the x direction (F_x_). Nanoparticles continue to move under the influence of liquid force in the y-direction without deviation in the x-direction. However, when the particle moves in the y-direction and reaches closer to the sub-channel graphene ribbon (GR_2_), the particles experience a gradient force in the x-direction exerted by the GR_2_, leading to deviation of a specific (larger) nanoparticle in the x-direction. Large particles sense stronger forces in the x direction, so the deviation of a large particle is more significant than the smaller ones. Therefore, the larger particle changes its path toward the GR_2_ and is filtered out from the main channel. Due to interaction with the GR_2_ (which is shown later) the F_x_ component of force felt by the nanoparticle became zero but it still has F_y_ to push the nanoparticles toward outlet.1.

It is worth mentioning, despite that sorting the larger particle in step.1 the smaller nanoparticles also felt force in the x direction and experienced a small deviation from the center of the main channel. Therefore, another graphene nanoribbon (GR_3_) is placed in the main channel to push back undesired deviation to the center of the main channel.

Likewise step.1 when the particles were stablished in the center of the main channel their F_x_ became zero but still move in the y direction due to liquid force. When the particles reach closer to GR_4_ the 5 nm radius particle which is the larger one after sorted 10 nm particles felt an immense -F_x_ and like 10 nm nanoparticles deviate from the main channel and sorted by GR_4_ and keep moving in the second subchannel toward the outlet.2.

In step 3 the nanoparticles which remained in the main channels are only 2.5 nm and smaller than 2.5 nm. therefore, the largest one deviated from its main path in the main channel and fall into the trap by GR_5_ in the x direction but likewise other nanoparticles it is free in the y direction and keep moving toward outlet.3.

In step.4 due to the very small size of the nanoparticles there is no need to establish another graphene nanoribbon in the main channel because their undesired deviation is almost zero, therefore the remained nanoparticles with very small sizes of R < 2.5 nm keeps moving toward to outlet. 4 by considering the main path end as another outlet to remained nanoparticles in the microfluidic sample int he main channel.

Another notable feature in this design is that as the particle size becomes smaller, the gap between the ribbon and the main channel becomes smaller until the small particle senses enough F_x_ to deviate from its current path. On the other hand, if this gap is too small, particle deviation becomes very large, and the ribbon cannot trap the particle; likewise, if the gap is too large, the particle ∆x will be negligible and moves in the main channel.

In the optical analysis, we consider a total field-scattered field (TFSF) plane wave source with a constant intensity of 0.6 mW/ µm^2^. This source is supposed to be polarized along the graphene surface with the propagation direction normal to the structure’s surface. Finite-Difference Time-Domain (FDTD) method and Perfectly Matched Layers (PMLs) are employed in all direction in our simulations. The simulation domain's height (along z) is 5 µm, and the plane wave source is placed 2 µm above the graphene surface.

**Figure 2:**
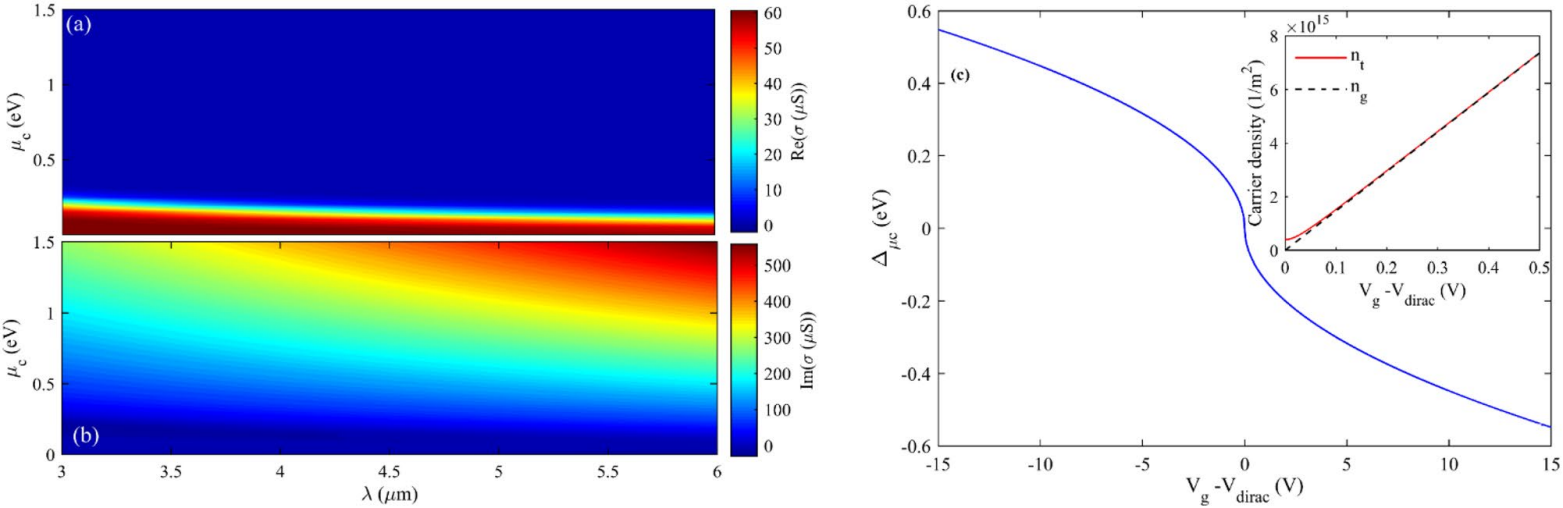
(**a**) Real and (**b**) imaginary parts of the surface conductivity of graphene versus wavelength and chemical potential (µ_c_), (**c**) variation of chemical potential vs. gate voltage, and the total and gate-induced charge carrier density at graphene surface.

Graphene is modeled as a two-dimensional sheet in FDTD analysis, and according to^45,47^, its surface conductivity is described by Random Phase Approximation (RPA) which is given by:

1 $$ \sigma \left( {\omega ,\mu_{c} ,\Gamma ,T} \right) = \sigma_{{{\text{inter}}}} + \sigma_{{{\text{intra}}}} = \frac{{je^{2} \left( {\omega - j2\Gamma } \right)}}{{\pi \hbar^{2} }} \times \left[ {\frac{1}{{\left( {\omega - j2\Gamma } \right)^{2} }}\int\limits_{0}^{\infty } {\varepsilon_{\sigma } \left( {\frac{{\partial f_{d} \left( {\varepsilon_{\sigma } } \right)}}{{\partial \varepsilon_{\sigma } }} - \frac{{\partial f_{d} \left( { - \varepsilon_{\sigma } } \right)}}{{\partial \varepsilon_{\sigma } }}} \right)d\varepsilon_{\sigma } } } \right.\left. { - \int\limits_{0}^{\infty } {\frac{{f_{d} \left( { - \varepsilon_{\sigma } } \right) - f_{d} \left( {\varepsilon_{\sigma } } \right)}}{{\left( {\omega - j2\Gamma } \right)^{2} - 4\left( {\varepsilon_{\sigma } /\hbar } \right)^{2} }}d\varepsilon_{\sigma } } } \right] $$

where* ħ* is the reduced Plank constant, *T* is the temperature, *ω* is the angular frequency, *μ*_*c*_ is the chemical potential, *Γ* is the phenomenological scattering rate that is assumed to be independent of energy *ε*_*σ*_*,* and *f*_*d*_* (ε*_*σ*_*)* is the Fermi–Dirac distribution function. Real and imaginary parts of the Surface conductivity of graphene versus chemical potential are illustrated in Fig. 2.

Like a parallel plate capacitor, charge carriers will be accumulated at the graphene sheet since a gate voltage is applied to Si as another plate of the capacitor. Charge carrier density induced by the gate voltage can be obtained by *n*_*g*_ = *C*_*g*_* (V*_*g*_*-V*_*dirac*_*)*/*q*, in which *C*_*g*_ is the gate capacitance, *V*_*dirac*_ is the applied gate voltage corresponding to *E*_*f*_ = 0 in graphene, *V*_*g*_ is the applied voltage to the gate and *q* is the unit charge. Accumulating charge carriers in the graphene shift the Fermi level so that this shift versus the gate voltage can be calculated via Eq. (2).

2 $$ \left| {\Delta E_{f} } \right| = \hbar \nu_{f} \sqrt {\frac{\pi }{q}\frac{{\varepsilon_{0} \varepsilon_{r} }}{{d_{ox} }}\left| {V_{g} - V_{dirac} } \right|} $$

In this equation, *ε*_0,_ *ε*_*r*_, and *d*_*ox*_ are the vacuum permittivity, dielectric permittivity, and dielectric thickness^46,48^, respectively. Figure 2c demonstrates the graphene chemical potential shift as a gate voltage function. The inset represents the graphene surface's total and gate-induced charge carrier density as one capacitor plate. In our previous work^19^ the electromagnetic analysis of graphene based devices using FEM method and the parallel plate capacitor model has been explained with more detailed.

**Figure 3:**
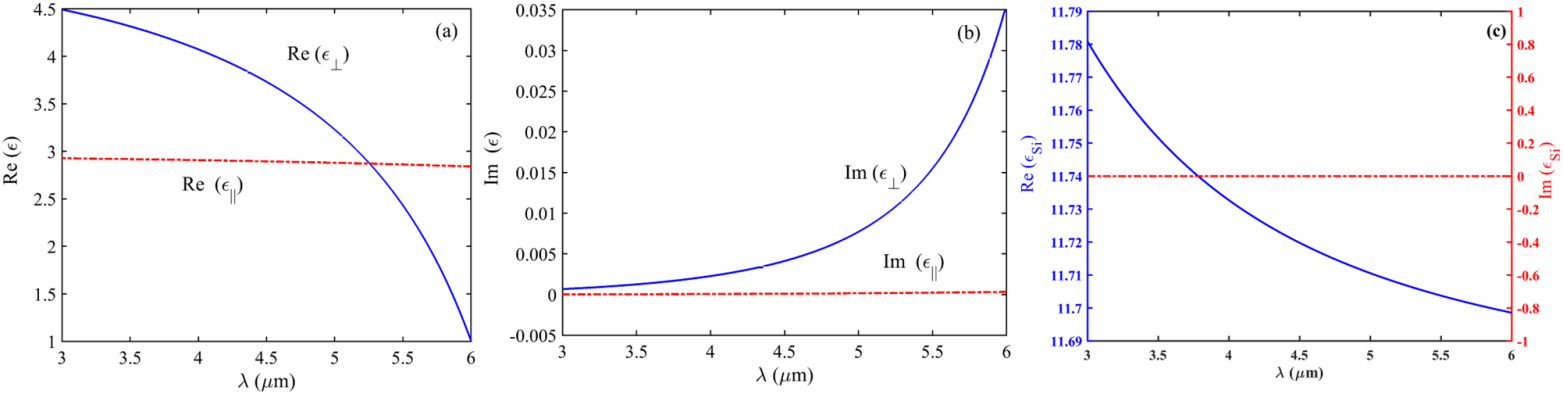
The real (**a**) and imaginary (**b**) parts of hBN anisotropic permittivity (**c**) The Real and imaginary parts of Si permittivity as a function of wavelength, respectively.

In the given nanoparticle sorter, the hBN is modeled as anisotropic bulk material with parallel and perpendicular real and imaginary parts of permittivity functions obtained from Eq. (3). This material is a van der Waals crystal with two kinds of IR active phonon modes relevant to its hyperbolicity^39^.

3 $$ \varepsilon_{m} = \varepsilon_{\infty ,m} \left[ {1 + \frac{{\left( {\omega_{LO,m} } \right)^{2} - \left( {\omega_{TO,m} } \right)^{2} }}{{\left( {\omega_{TO,m} } \right)^{2} - \omega^{2} - j\omega \Gamma_{m} }}} \right] $$

where *m* = ⊥, //, ϵ_∞,⊥_ = 4.87, ϵ_∞,∥_ = 2.95, Γ_⊥_ = 5 cm^−1^, and Γ_∥_ = 4 cm^−1^^42,43,49,50^. The real and imaginary parts of hBN anisotropic permittivity as a function of the wavelength range are also illustrated in Fig. 3. The imaginary part of permittivity in both directions is almost zero. Therefore, hBN acts as a lossless dielectric material in this wavelength range and a perfect substrate for graphene ribbons. Out-of-plane phonon modes with ω_TO_ = 780 cm^−1^ and ω_LO_ = 830 cm^−1^, and in-plane phonon modes with ω_TO_ = 1370 cm^−1^, ω_LO_ = 1610 cm^−1^ (ϵ⊥ < 0, ϵ∥ > 0) are extracted from^42^. Also, the permittivity of the Si is taken from^51^ and illustrated in Fig. 3c. As it is clear in the range of 3-6 µm wavelength the real part of its permittivity changes only 0.1 and its imaginary part is always about zero.

The graphene-hBN stack can be created by dry-transferring graphene over thick mechanically cleaved hBN crystal, as described in^52^. To fabricate graphene nanoribbon on an hBN surface a mechanical transfer method^52^ to deposit graphene on hBN substrates can be used which is a special method to transfer graphene from one substrate to an exact location on another substrate (in this case an hBN flake). this method can be used to fabricate multilayer graphene nanoribbons^53^. In this method, extreme care is taken to reduce water residues and this is named the “dry transfer method”. They adopted the dry transfer method to our needs. A dry transfer method consists mainly of four steps as illustrated In step A the PMMA (carrying the graphene) is suspended in a plastic window that has been attached to the chip by using double-sided tape to the PMMA side. Next in step B, sacrificial PVA film is dissolved in DI water to release the PMMA film from its substrate. The graphene is on top of the PMMA so it will never be in contact with water. In step C they adhered the PMMA membrane to an aluminum transfer slide, which is clamped to a mask aligner modified. During the transfer process, they fix the hBN-containing substrate on the holder. In step D, using the optical mask micro-manipulators, they align the graphene flake to the hBN crystal and lower the polymer side onto the substrate. When the polymer touches the substrate, it makes strong contact with the SiO_2_ substrate. Then, they turn off the nitrogen valve of the mask aligner and down the holder substrate along with the PMMA polymer attached to it. They finally put the substrate with target hBN flake on a custom-made heater that they set to 110˚C to enhance adhesion between graphene and hBN for at least 10 min. Next, the sample is allowed to cool down and the PMMA is removed with acetone and isopropanol (IPA). As chemical cleaning with organic solvents always leaves some residues, they anneal the samples as well. At this stage, they heat the samples slowly to 400˚C in a tube oven in forming a gas environment and anneal them there for ∼3 h before slowly cooling them down to room temperature. This treatment is very effective in removing polymer residues from the transfer.

Since this fabrication method requires only one cleaning step, it allows for fast device preparation of graphene on hBN with little bubbles and wrinkles. Electrical transport measurements of the graphene devices on hBN have mobilities and carrier inhomogeneity that are almost an order of magnitude better than devices on SiO_2_.

The principle of optical tweezers is based on the observation that light possesses momentum. Scattering force and gradient force are the two most frequent forms of optical forces. The scattering force results from photonic momentum conversion, whereas the gradient force arises when the light field's distribution is nonuniform. However, in Rayleigh particle (a⟪λ) the scattering force is negligible. In order to evaluate optical forces acting on a particle at first, we integrate MST on the surface of the particle^54^. The exerted average optical force is given by:

4 $$ F = \frac{1}{2}\oint {(\left\langle {T_{M} } \right\rangle \cdot } \hat{n})dS $$

where n is the normal unit of the pointing vector outward to the surface *S* enclosing the particle, and *T*_*M*_ is the Maxwell stress tensor obtained by Eq. (5):

5 $$ T(r,t) = \varepsilon_{T} E(r) \otimes E^{*} (r) + \mu_{T} H(r) \otimes H^{*} (r) - \frac{1}{2}\left( {\varepsilon_{T} \left| {E(r)} \right|^{2} + \mu_{T} \left| {H(r)} \right|^{2} } \right) $$

where *ε*_*T*_ and *µ*_*T*_ represent the medium permittivity and permeability, and **E** and **H** are the electric and magnetic field intensity vectors. *r* and *t* represent the position vector and time, respectively.

When the particle is guided by the evanescent field and moves along a planar substrate in a flow channel and is pushed by a scattering force depending on its radius and distance from the substrate, it experiences the Stokes drag^55^, which is described by:

6 $$ F = 6\pi \mu_{f} a\nu \left( {1 - \frac{9}{16}\frac{a}{h} + \frac{1}{8}\left( \frac{a}{h} \right)^{3} - \frac{45}{{256}}\left( \frac{a}{h} \right)^{4} - \frac{1}{16}\left( \frac{a}{h} \right)^{5} } \right)^{ - 1} $$

where *µ*_*f*_*, v, a,* and *h* are the viscosity of water taken to be 0.89 mPa.s at the room^56^, the particle velocity, particle radius, and the distance between the surface and the center of the particle, respectively.

## Results and discussions

**Figure 4:**
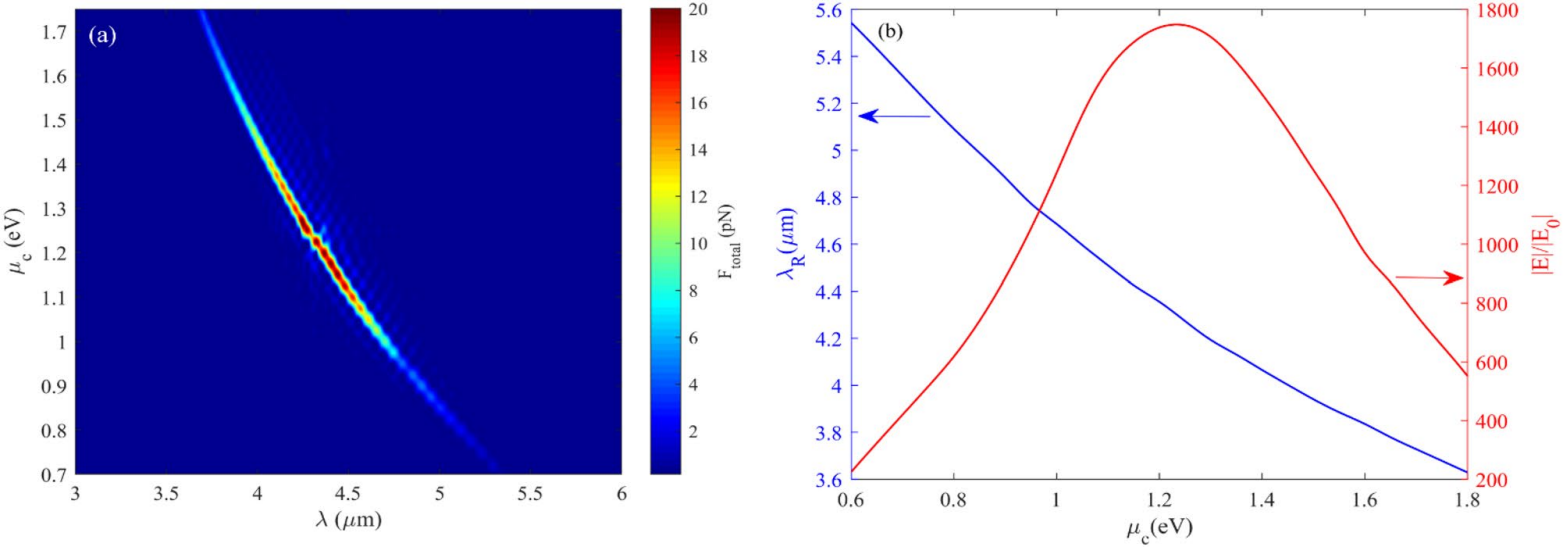
(**a**) The total force felt by a particle with a 20 nm radius as a function of graphene’s chemical potential and wavelength of the incident light (**b**) electric field enhancement and resonance wavelength variation versus graphene’s chemical potential.

As discussed earlier, an increment of the graphene Fermi level makes the plasmonic effect more robust and enhances the electric field at the surface of the ribbon. Figure 4a represents the total force felt by a 20 nm particle versus graphene’s chemical potential and the wavelength of the incident light. It is clear that the total force reaches its maximum value in the wavelength range of 4–4.5 µm and the graphene chemical potential of 1–1.3 eV; the maximum force felt by any particle happens at a resonance wavelength of 4.26 µm, and graphene’s chemical potential of 1.15 eV. Figure 4b illustrates the electric field enhancement and the resonance wavelength variation as a function of graphene’s chemical potential. The electric field magnitude at the resonance wavelength of 4.26 µm becomes 1800 times larger than the source electric field.

According to Fig. 4b, at the graphene’s chemical potential of around 1.2 eV, the electric field reaches its maximum value at the surface of the graphene ribbon (Cross section). However, since the graphene's chemical potential increases to 1.25 eV, the electric field decreases until it reaches zero, and the graphene’s optical properties disappear. Hence there is not any graphene ribbon to affect nanoparticles.

**Figure 5:**
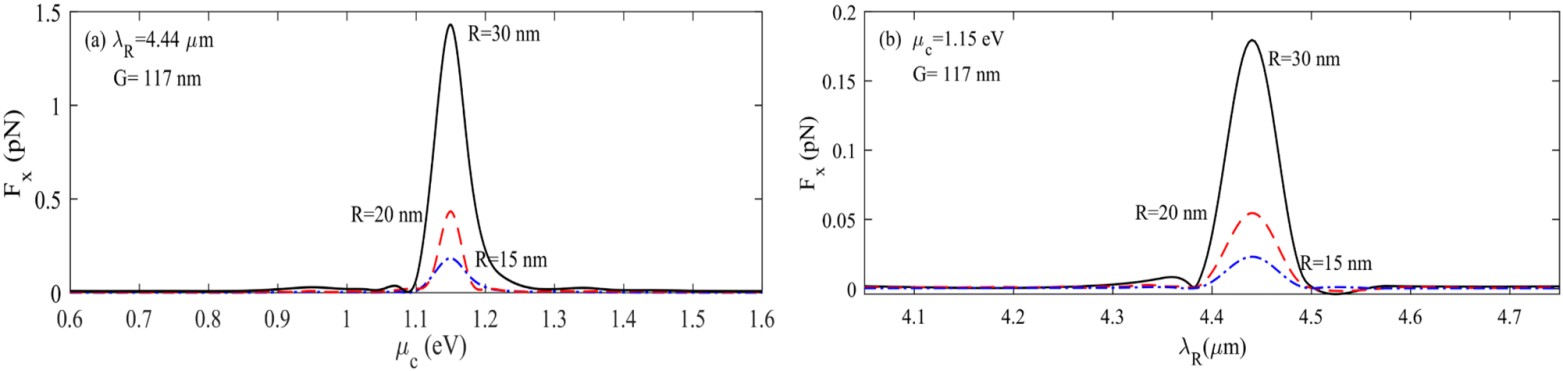
F_x_ of particles with R = 15, 20 and 30 nm at (**a**) λ = 4.26 µm and µc between 0.8 and 1.7 eV and at (**b**) λ_R_ between 3.8 and 4.7 µm and µc = 1.25 eV.

Here, we suppose a structure with only two channels for analyzing the sorter's function. One of these channels is the main and, and the other is located at the substrate with a definite gap and their width are 120 nm in x-direction. Figure 5a demonstrates the F_x_ exerted on nanoparticles of different sizes, which are fixed at the center of the main channel, where the graphene ribbon in the second channel has a 117 nm gap from the main channel. Larger particles experience a more significant force. In Fig. 5a, for particles with R = 15, 20, and 30 nm, F_x_ is calculated for the constant source wavelength of 4.44 µm, which is the plasmonic resonance wavelength of graphene ribbons. For nanoparticles with different sizes, the value of µ_c_ is constant. Figure 5b represents the exerted force to the same nanoparticles versus incident light wavelength at a fixed graphene’s chemical potential of 1.15 eV. One can see that the electric field confinement reaches its maximum value when the incident light wavelength is 4.44 µm. This figure shows that the exerted force on the particles highly depends on the size of these particles, but the resonance wavelength does not vary with particle sizes.

**Figure 6:**
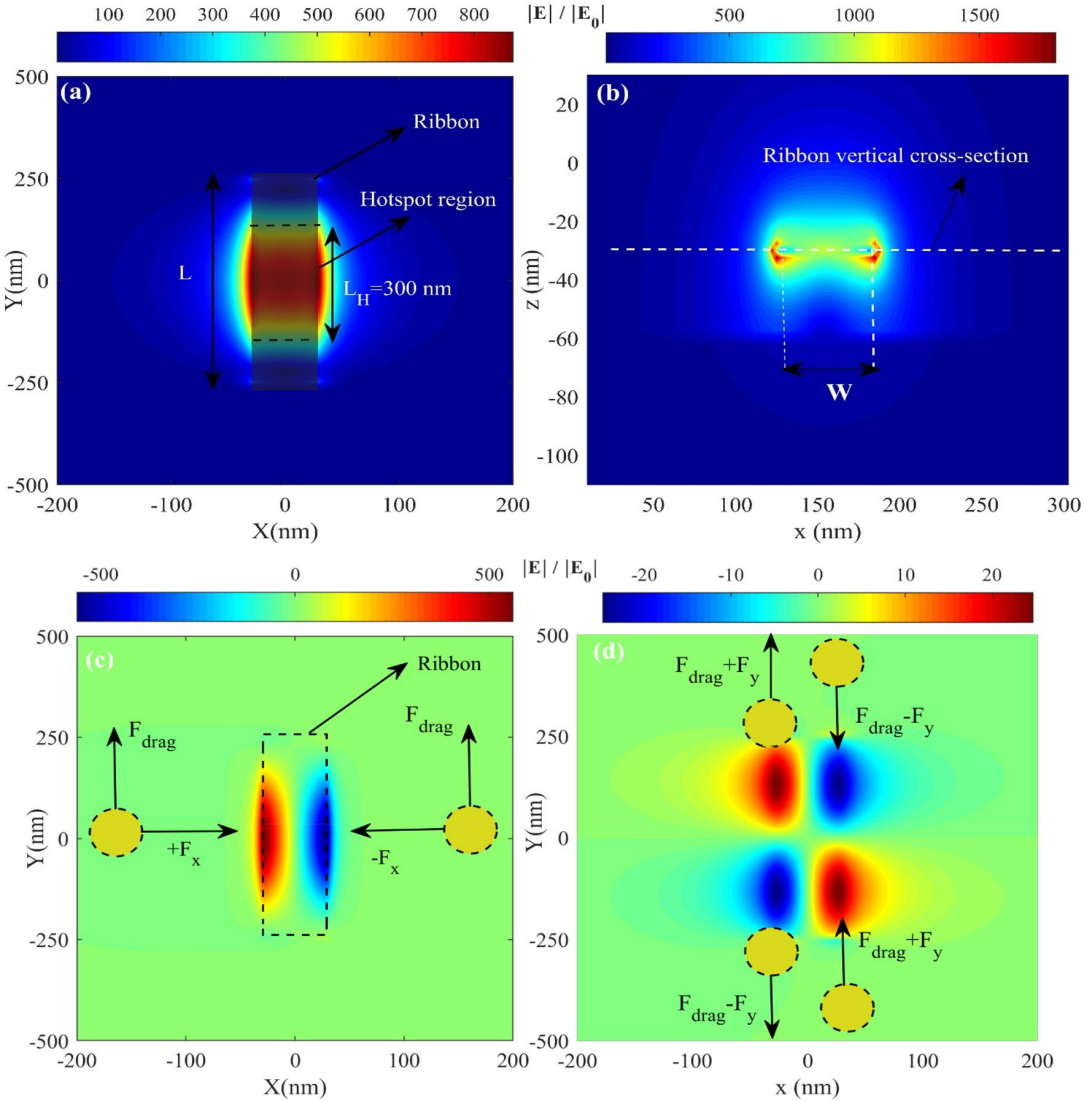
Represents the total component of the electric field at the top view (**a**), the cross-section view of the total electric field (**b**), the x component of the electric field from the top view (**c**), and the y component of the electric field from the top view (**d**).

Figure 6a represents the total electric field on the graphene surface, and Fig. 6b depicts the total electric field on the xz surface of the graphene ribbon. The electric field at the sideline edges significantly increases to 1500 folds. The graphene ribbon is in the on-state at the resonance wavelength. As shown in Fig. 6a,b, the field gradient at both center of the ribbon and two lateral edges in its middle is higher than other parts and reaches its maximum value at two sides of the ribbons. Hot spots of the electric field are formed at the edges of the ribbon. Figure 6c,d represent x and y components of the electric field profile along with the optical force direction in various positions. It can be seen that the y component of the electric field is too small than the x component. Thus, the particle experiences a smaller optical force along the y-direction, which can be ignored compared to other components of the optical force. The reason is that the source is x polarized, perpendicular to the graphene subsurface.

**Figure 7:**
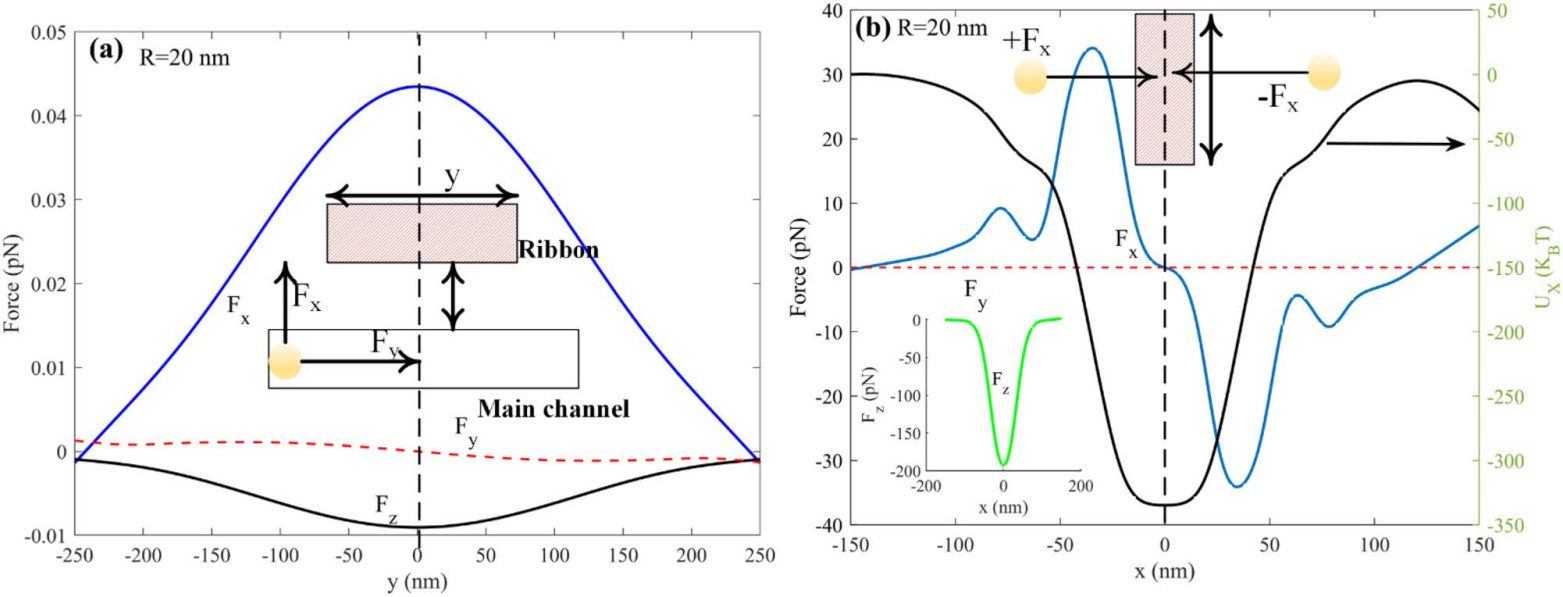
Forces felt by an R = 20 nm particle when (**a**) it is fixed at x = 0 and moves along the channel with G = 117 nm and (**b**) it is fixed at y = 0 and moves through the width of the channel with G = 117 and its potential of trapping.

Figure 7b represents the induced optical force on the 20 nm particle in all directions when moving perpendicular to the graphene ribbon. As it can be seen, the more particle moves toward the nanoribbon, the more significant force it feels. Fx has two peaks when the nanoparticle reaches two sides of the graphene nanoribbon; meanwhile, at the center of the ribbon, Fx is zero due to the symmetry of the structure. When the particle crosses the nanoribbon, Fx is negative and pulls the particle back toward the center of the ribbon, where it is trapped. In the case of Fz, it is always an opposing force pulling the nanoparticle to the surface of the structure, and unlike the Fx, it has a maximum amount at the center of the ribbon. Moreover, as shown in Fig. 7a, when the nanoparticles move along the y-direction with a constant gap from the ribbon at fixed x = 0, F_x_ and F_z_ have maximum values at the center of the ribbon. In addition, F_y_ is almost negligible in the y-direction. It is worth mentioning that here the dynamic analysis is not investigated yet, and the figures show that particles of any size can be trapped by nanoribbons. The dynamic state of the proposed system will be vastly investigated in the next section.

**Figure 8:**
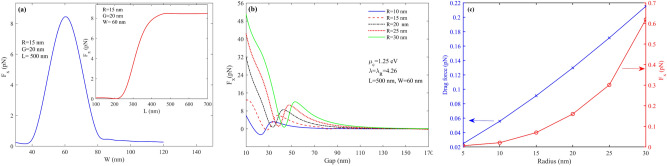
F_x_ for (**a**) an R = 15 nm particle L = 500 and w between 30 and 120 and w = 60 nm and L between 100 and 700 (**b**) R = 10 to 30 nm and G between 10 and 170 nm (**c**) drag and F_x_ for particles with R = 5,10,15,20,25 and 30 nm.

The gap between nanoparticles and graphene ribbon significantly affects the optical force experienced by nanoparticles moving in the main channel. The exerted force increases by decreasing the gap between them. Figure 8b illustrated the proportion of gap and exerted F_x_ for five various nanoparticles when they are fixed at x = y = 0. This figure shows that the force decreases by increasing the distance between the nanoparticle and ribbon from 10 to 170 nm. Furthermore, the width and length of the graphene nanoribbon can impose an electric field enhancement such that the exerted force of the nanoparticle is affected, which is shown in Fig. 8a. by calculating the force of the nanoparticle with a radius of 20 nm for widths between 30 and 120 and lengths 100 to 700, it can be seen that the higher force is achieved at width = 60 nm and length above 500. Furthermore, it can be seen that at W = 60 nm, F_x_ exerted on the nanoparticle has the maximum value in the x-direction. In addition, particle size and optical forces can vary the drag force felt by nanoparticles induced by microfluidic flow in the inlet. Figure 8c represents the relation between the drag force with particle size and the x component of force felt by the nanoparticle; here, the gap is fixed at 117 nm. Both forces increase by increasing the particle size, and FX variations with particle size allow sorting particles based on their size.

We have statically investigated the parameters that affect the manipulation of nanoparticles. In the following, dynamic structure simulations are evaluated, giving us more exciting information about the designed structure. Here we investigate the particle's movement along the channel regarding time for differing gaps and sizes. Since the gap variations affect the induced F_x_ on a nanoparticle, it experiences a different trajectory. However, when the gap is fixed to its optimized value, the nanoparticle is trapped above the ribbon. In contrast, nanoparticle keeps moving at the main channel at large gaps.

Figure 11 represents the dynamic simulation of the trapped particle with a radius of 10 nm and gap = 117 nm. The x component force when the particle reaches the ribbon area has positive and negative values. The particle has no movement in the x direction when the induced force sign is changed. However, as the particle moves forward by a drag force, F_x_ increases, and the particle deviates from the main channel and moves toward the ribbon. At the small gap, F_x_ reaches a high value.

**Figure 9:**
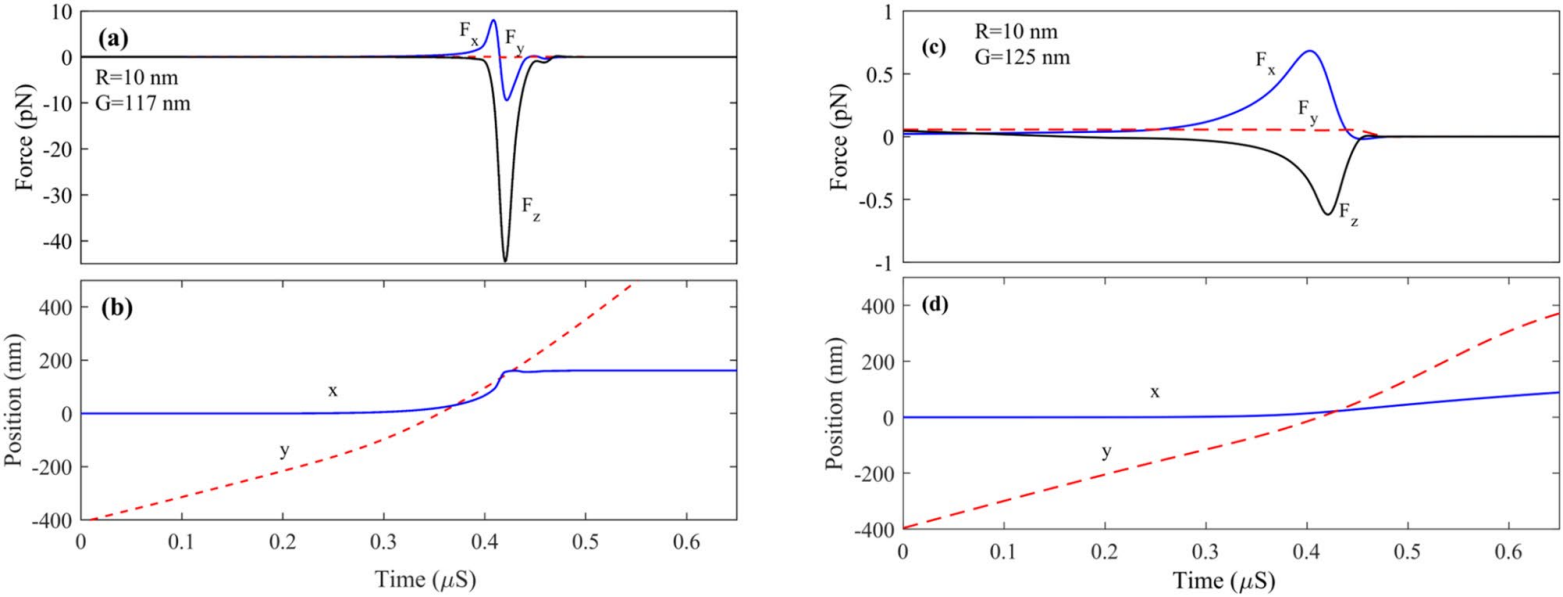
(**a**, **b**) Force and position as a function of time for a nanoparticle with R = 10 nm and G = 117 nm, respectively. (**c**, **d**) Force and position as a function of time for a nanoparticle with R = 10 nm and G = 125 nm respectively.

The particle passes through the ribbon due to the particle's momentum, similar to Fig. 9b. The force pulls back the particle toward the ribbon; this movement continues until the force is damped and remains constant at zero. By comparison, Fig. 11a,b, which show the force and position of the particle at the time domain, can be realized around 0.36 um, which F_x_ has damped the x-direction movement of the particle, and it became constant. In other words, the ribbon electromagnetic field traps it. After the particle is trapped, it moves along the y-direction concerning the drag force shown in Fig. 8. Its x-direction becomes fixed to 161 nm since no force is exerted on the particle in x direction, which leads to sorting this specific radius from a smaller radius at the main channel. Figure 9c,d represent the same particle, however, with a larger gap of G = 125 nm. As shown at 0.4 µS, F_x_ has the maximum positive value, which means the particle felt force in the + x direction. However, due to the large gap and low exerted force on the particle, the particle's position has no considerable change in the x-direction and keeps moving in the main channel.

Notably, when the larger particle is sorted, the smaller particle deviates from its main path. However, due to minor F_x_ in comparison to the trapped particle, it remains at the main channel, as shown in Fig. 9. To pull back the deviated undesired particle from the center of the main channel; as can be seen in Fig. 1b, we set another ribbon at the center of the main channel which causes the all-unsorted particle fixed again at the center of the channel.

**Figure 10:**
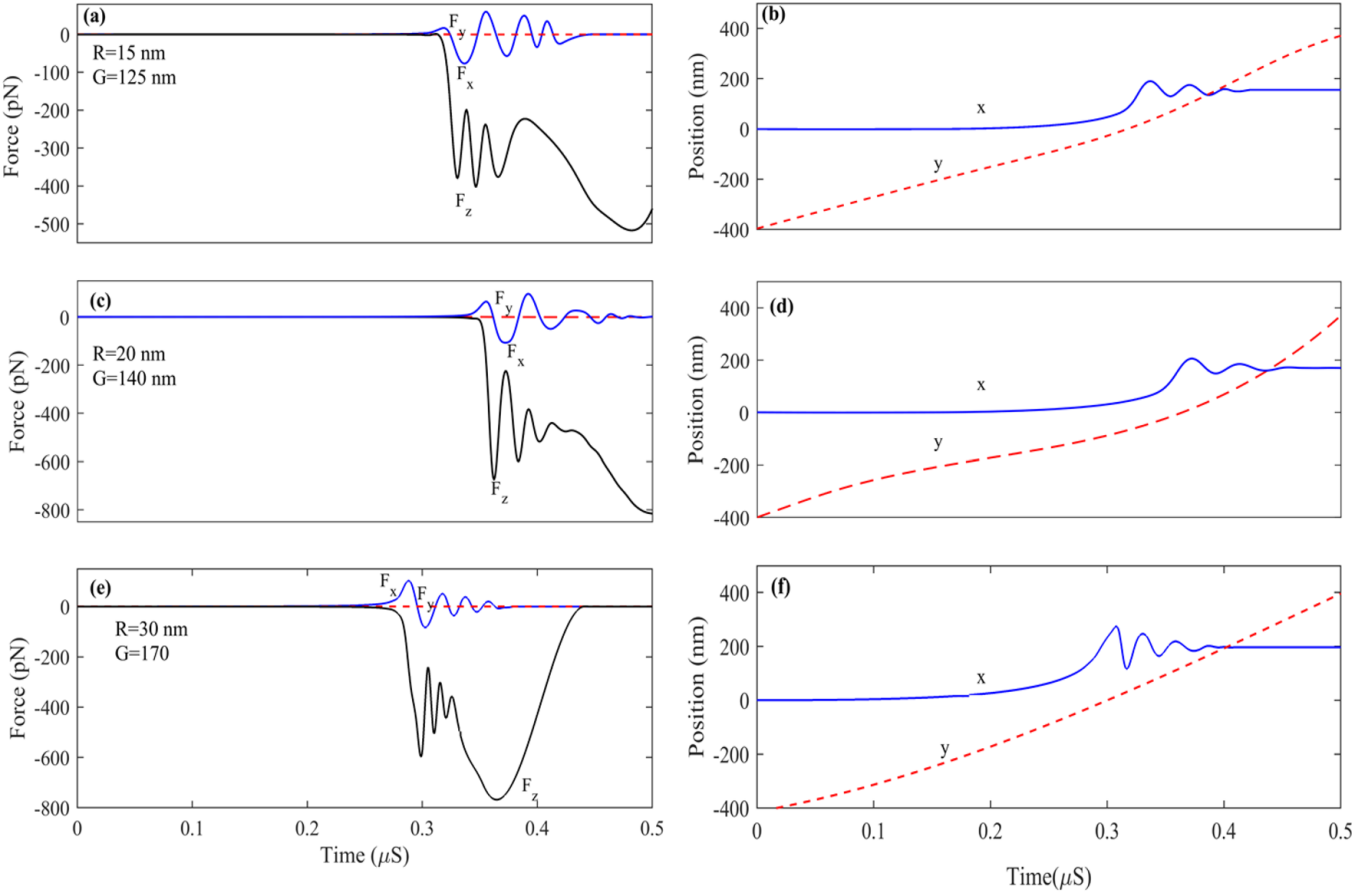
Force and position for nanoparticles with (**a**, **b**) R = 15 nm and G = 125 nm. (**c**, **d**) R = 20 nm and G = 140 nm. (**e**, **f**) R = 30 nm and G = 170 nm.

Figure 10a–f represents the time-domain simulation of larger nanoparticles with 15, 20, and 30 nm radii. As can be seen from Fig. 12a, the 15 nm particle is wholly trapped at the center of the ribbon where x = 155 nm with G = 125 nm; in this case, due to the larger size particle compared to R = 10 nm, F_x_ takes more time to reaches steady-state, in another word particle 2 or 3 times path the ribbon until it trapped at the center of the ribbon according to Fig. 10a particle after 0.45 µs reaches to steady-state and trapped at the center of the ribbon. Although Fz always has a negative value, it experiences remarkable variation in time. The reason is that at the ribbon center F_z_ reaches its maximum value therefore F_z_ peaks each time the particle paths the center of the nanoribbon. Figure 10c,d represent the dynamic response of particles with R = 20 nm; as it can be seen, after t = 0.48 µs, the particle reaches a steady state and is trapped at the center of the ribbon x = 170 nm. Figure 10e,f represent the dynamic response of particles with R = 30 nm when G = 170 nm. As can be seen by comparing particles with radius 20 and 30 nm, the larger particles take more time to be damped, and the number of times it passes through the ribbon to reach the damping state is more. It can be seen from Fig. 10f particle at t = 0.4 µs trapped at fix x = 200 nm.

**Figure 11:**
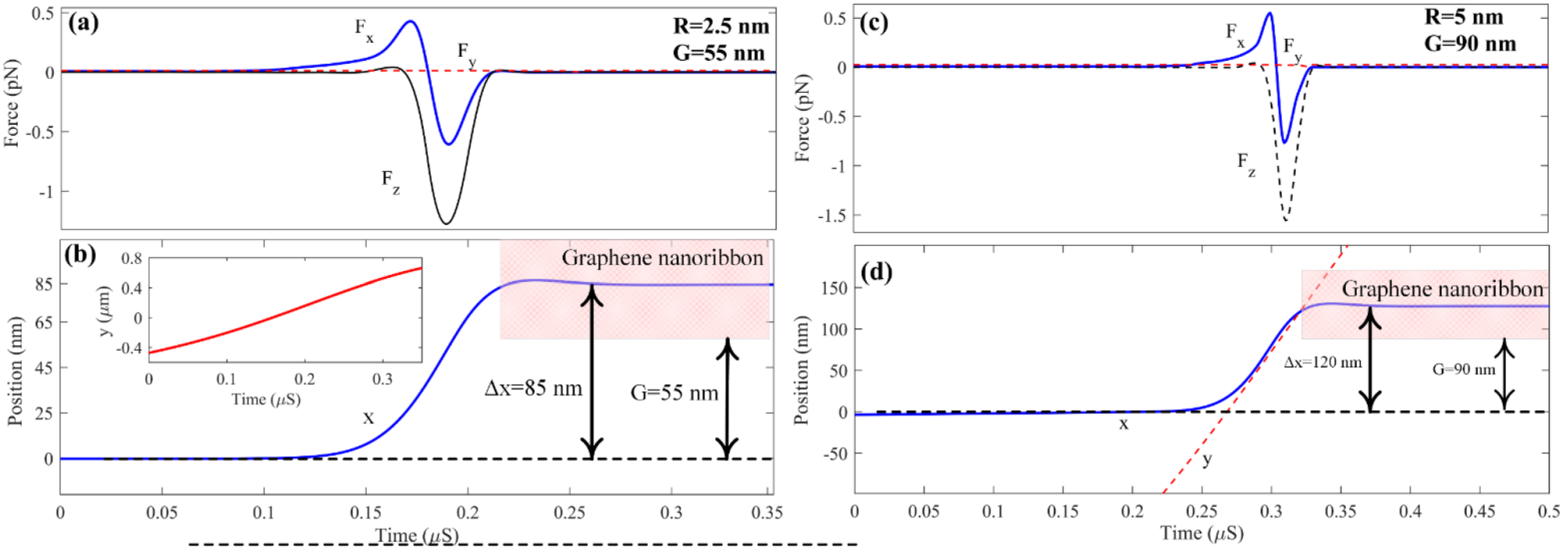
Force and position versus time for nanoparticles with (**a**) and (**b**) R = 2.5 nm and G = 55 nm and (**c**), (**d**) R = 5 nm and G = 90 nm.

One of the notable features of this structure is sorting tiny particles. As shown in Fig. 11, the particles with radii 5 and 2.5 nm are trapped when the ribbon is fixed to G = 90 nm and G = 55 nm, respectively. By sorting 2.5 nm nanoparticles at the main channel, only the particles with radii smaller than 2.5 nm remain and move toward the outlet of the main channel. Figure 11 shows how small nanoparticles can be quickly sorted by making ribbons closer to the main channel. As discussed above, the small nanoparticles felt a small force compared with larger ones. Hence to desire to sort the small particles, the ribbon must be fixed at small gaps. As can be seen in Fig. 11a, the particle with a radius of 2.5 nm can be deviated from its main path and trapped at the center of the ribbon where x = 85 nm by fixing the ribbon with G = 55 nm. the particle, as discussed above, moves faster in comparison to larger particles. Therefore, it is trapped very soon at t = 0.215 µs. After this time, the x component of the particle becomes fixed, and the y component keeps moving toward a more extensive value until it reaches the edge of the structure and its outlet. Likewise, the 5 nm particle dynamic response shown in Fig. 11c,d, As can be seen in the smaller particles, force as a function of time has only one sinusoidal pattern; nevertheless, sinusoidal behavior is no longer observed in the position diagram in terms of time which are shown in Fig. 11a,b respectively. The reason is that tiny particles have small masses; therefore, the momentum of this article is also tiny to overcome the particle velocity on the ribbon and trap it easier. The 5 nm particle is trapped when the ribbon gap is fixed to G = 90 nm at its x component at 125 nm after t = 0.32 µs.

**Figure 12:**
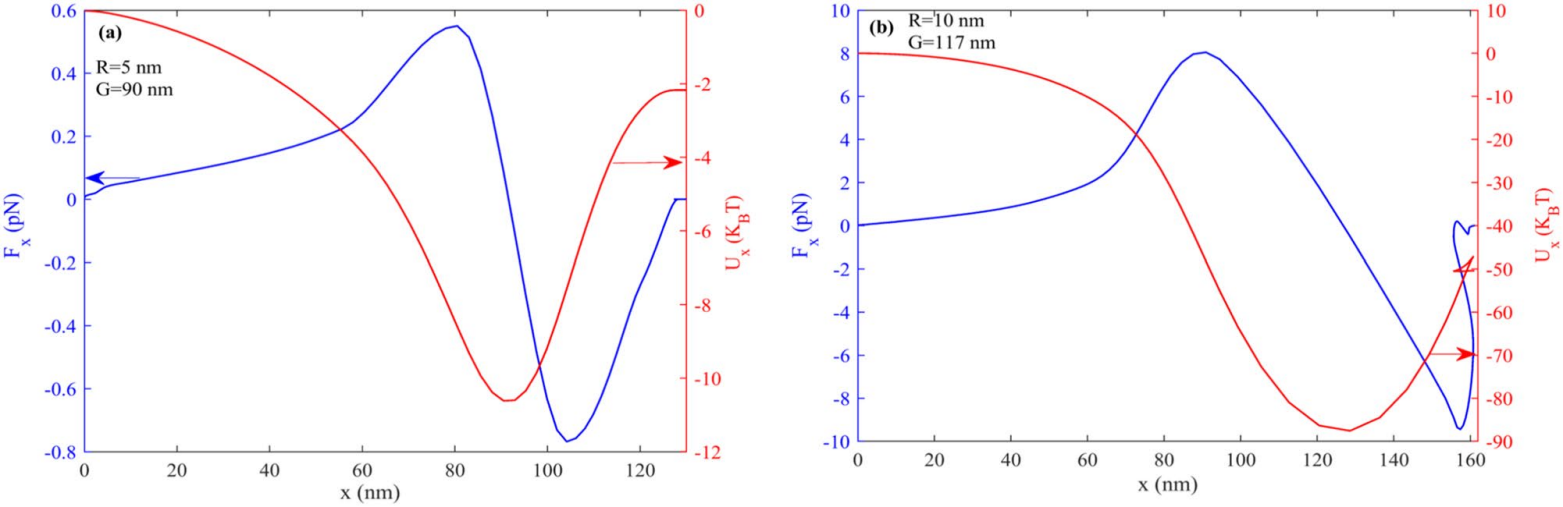
Force and potential of trapping versus x for nanoparticles (**a**) R = 5 nm and G = 90 nm (**b**) R = 10 nm and G = 117.

Figure 12a,b represent the nanoparticle's force and trapping potential energy in the x-direction for R = 5 nm and R = 10 nm, respectively. As can be seen, the trapping potential energy is higher than 10 K_B_T for both particles. On the other hand, the potential chemical peaks at around 120 and 90 nm, equal to the G of both particles, which means the trapping potential has the maximum value at the left side of the ribbon. Likewise, the force change sign at potential chemical peaks means that when the particle path on the left side of the ribbon F_x_ tries to pull back to the ribbon, it takes more negative force to overcome its initial momentum due to the particle velocity. It is clear from both figures that the negative sign of force is larger than the positive sign, resulting from the initial momentum of the particles.

**Figure 13:**
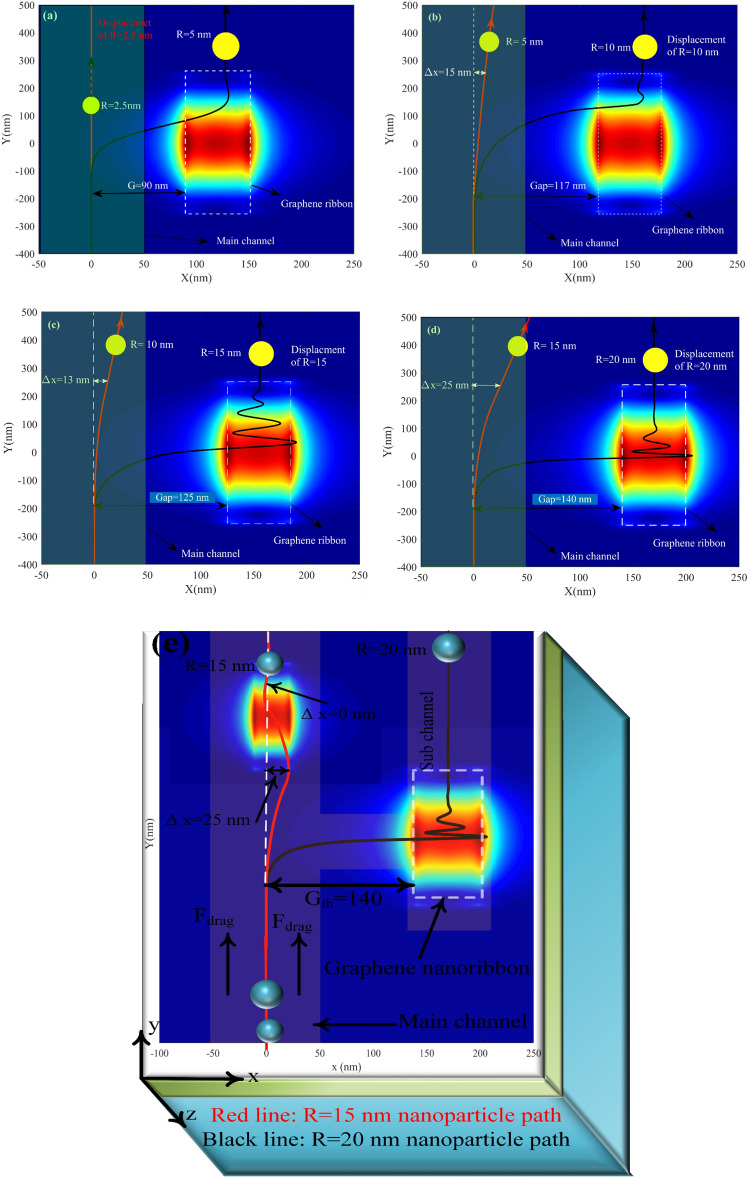
Displacement of nanoparticles with (**a**) R = 2.5 and 5 with G = 90 nm (**b**) R = 5 and 10 with G = 120 nm (**c**) R = 10 and 15 with G = 125 nm (**d**) R = 15 and 20 with G = 140 nm and (**e**) represents the dynamic response of R = 15 and 20 nm as same as (**d**) when the graphene nanoribbon located in the main channel is present.

Figure 13 indicates the nanoparticles' path along time at the threshold gap with the 5 nm smaller particle displacement from trapped particles. As shown in Fig. 13a, the particle with R = 5 deviated entirely from the main channel. In contrast, the smaller one with R = 2.5 nm has negligible displacement. Nevertheless, when the particle size becomes more prominent, the displacement of the smaller ones also becomes large. It is shown in Fig. 13b, in which the particle with R = 10 nm deviated from the main channel, but the 5 nm particle displacement is about 15 nm. However, it remains at the main channel, and at the next step, the ribbon put on the channel path pulls it back to the center. Figure 13c represents the path in which deviated R = 15 nm nanoparticle moves along time. The minor particle deviation is not enough to change its x component considerably the displacement of the undesired particle, which for a 10 nm particle, is only 13 nm. Likewise, in Fig. 13d, the path in which particle with R = 20 nm moves is shown, and the displacement of the undesired deviated particle is 25 nm and remain in the main channel.

As shown in Fig. 13d, although R = 20 nm has been sorted from the main channel by fixing the G_th_ to 140 nm, the smaller particle which has a radius of R = 15 nm owns an undesired deviation of Δx = 25 nm, and as can be seen, it reaches to the edge of the main channel, which is not desirable. Figure 13e represents how the graphene located on the main channel fixes this problem. Therefore, the graphene nanoribbons' functionality in the main channel can be seen clearly here which it prevents moving R = 15 nm nanoparticles in the unwanted devotion path. (please see supplementary information Gif. 3).

**Figure 14:**
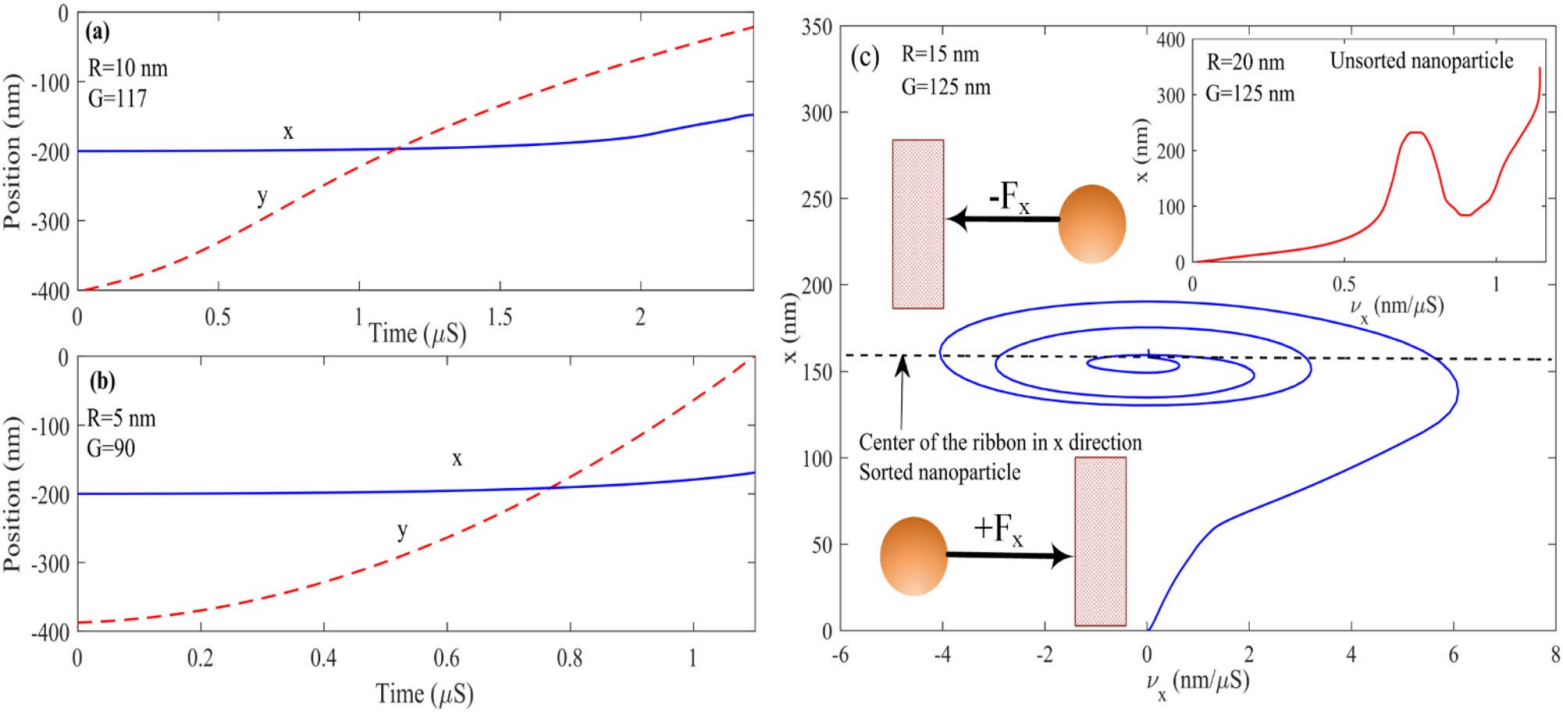
Displacement of nanoparticle with (**a**) R = 10 and G = 117 (**b**) R = 5 and G = 90 when the RI of nanoparticle is n = 1.5. (**c**)Velocity of nanoparticle with R = 15 nm and R = 20 nm with G = 125.

We analyzed the impact of nanoparticles' refractive index (RI) variation in manipulation. by decreasing the nanoparticle's RI, the differential between medium RI and nanoparticle decreases, which leads to a weak electromagnetic field and so that the exerted force on the nanoparticle decrease. as Fig. 14a,b indicates when RI changes from 1.57 to 1.5 the threshold gap cannot be able to trap the nanoparticles. Besides this, Fig. 14c illustrates the trapping mechanism for particles with radii of R = 20, 15 nm, and g = 125 nm. In this figure, the x-axis is the x component of the particle velocity, and the y-axis is the x component of the particle position vector. Initially, when nanoparticles R = 15 nm are located at the center of the channel, a positive force increases the particle velocity, moving it to the edge of the ribbon center. However, once the particle reaches x = 125 nm, the direction of force reverses, which causes the pulling of the nanoparticle instead of pushing it and decreasing particle velocity. When the nanoparticle passes x = 190 nm (out of the ribbon in the x-direction) due to negative F_X_, it returns to the left of the ribbon, and the nanoparticle experiences a negative velocity. Eventually, the speed direction reverses, and particles fall into the trap in a sinusoidal pattern by repeating these steps; the particle with R = 15 nm and G = 125 nm is damped, and its velocity reaches zero as expected at the center of the ribbon. In contrast, the inset of Fig. 14c depicts an unsorted particle due to a large nanoparticle. As shown, the particle experienced one-step fluctuation; however, in the second step, due to the high momentum of the R = 20 nm particle, it escapes from the range of ribbon effect and its electromagnetic field toward the right hand of the ribbon and large x. Therefore, this particle's x position cannot be fixed and goes out of the sub-channel. To see the animation form of the dynamic response of Fig. 13a–e see supplemental information.

**Figure 15:**
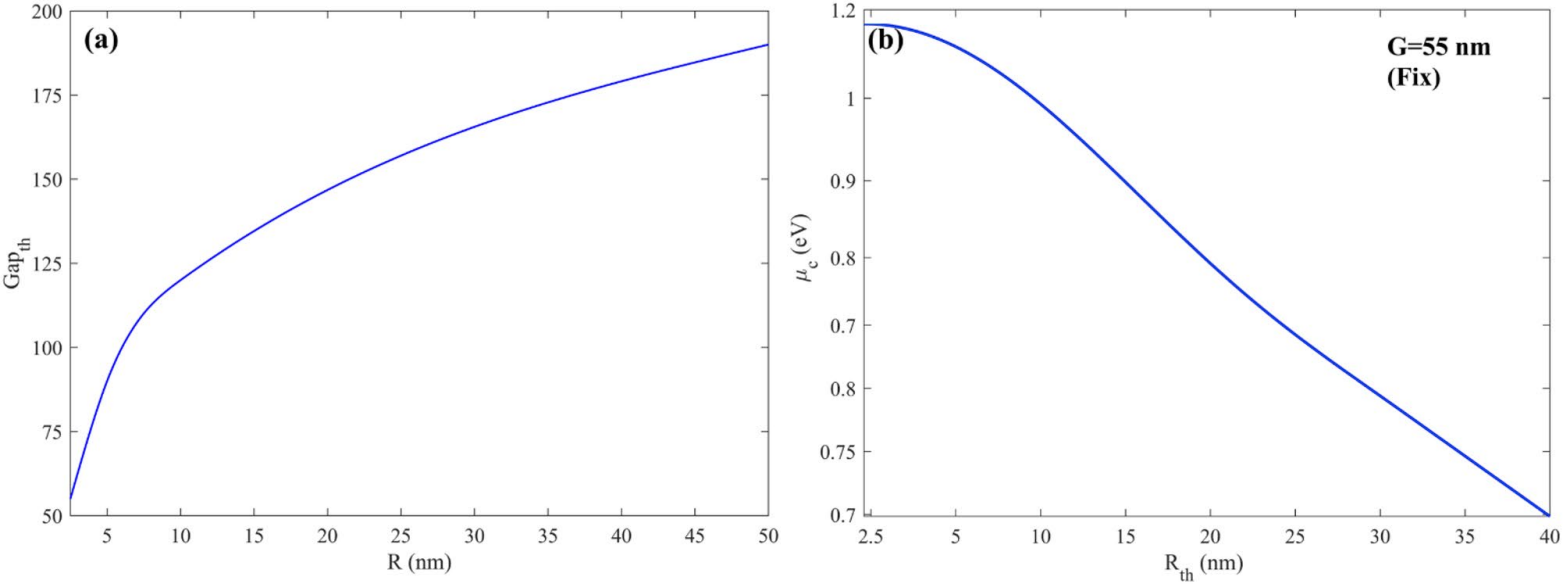
Threshold gap (**a**) and threshold chemical potential of graphene nanoribbons (**b**) as a function of sorted nanoparticle with radii 2.5–50 nm.

Finally, the threshold gaps between various particle sizes are explored. Figure 15a shows the end outcome. This study considers parts with a radius of 2.5–30 nm. As previously stated, smaller particles require smaller gaps to be trapped stably, whereas larger particles require larger gaps. Also, how many a nanoparticle close to the ribbon witnessed a larger electromagnetic field and, as a result, higher forces.

As we claimed at first, the proposed structure can also sort nanoparticles only with one subchannel or nanoribbon at a constant gap. The physical mechanism is the same as the threshold gap examined previously; however, instead of changing the gap to control exerted force on the nanoparticles, the shift in graphene chemical potential by applying a gate voltage to the Si substrate has been used to control exerted force for each nanoparticle. To clarify the algorithm for sorting particles with fermi energy, Fig. 4a,b can be helpful. As is evident in Fig. 4a, the optical force experienced by nanoparticles has a high dependency on graphene Fermi energy. This attitude of the proposed structure gives another flexibility to control the optical force evident by nanoparticles actively. As shown in Fig. 4a, the optical force felt by nanoparticles reaches its maximum value at µ_c,_ about 1.15 eV. Nevertheless, the optical force dropped sharply by increasing or decreasing µc from 1.15 eV. It means that a minor change in graphene chemical potential results in changes in optical force felt by nanoparticles. Therefore, the proper and suitable graphene chemical potential can be obtained by adjusting the biased gate voltage. Figure 2c represents the graphene nanoribbon chemical potential embedded in hBN (realistic value) as a function of applied gate voltage. Figure 15b represents the threshold graphene chemical potential as a function of wavelength in various nanoparticle sizes when the gap between the main channel and sub-channel is fixed at 120 nm. The threshold chemical potential of graphene to trap various size particles is represented in Fig. 15b.

By analyzing the threshold gaps and the graphs obtained from them, an equation can be proposed for both methods stated in this article, in which only with an error of less than 2.5 nm, the threshold gap and the potential energy of the graphene threshold for sorting all obtained nanoparticles with the desired radius. But not only for particles with a certain radius such as 30, 20, 15, etc. Rather, for any desired radius, one can easily find the threshold gap as well as the threshold form for sorting that specific radius. Equations 7 and 8, respectively, are given below to obtain the threshold gap for any arbitrary radius R and the threshold form for any arbitrary radius R.

7 $$ G_{th} \left( R \right) = \, a \times R^{b} + c $$

In Eq. 7, G_th_ represents the threshold gap shown in Fig. 1b for a specific gap radius of R. the value of a, b and c are constant and are equal to a = 1717, b = 23 × 10^–3^ and c = −1695.

Moreover, to the gap threshold as shown in Fig. 15b the proposed structure can sort the nanoparticles based on graphene nanoribbons’ chemical potential at fixed gaps. The following Eq. 8 represents the fermi level for sorting and deviation of any specific nanoparticles with radii of R.

8 $$ \mu_{{c_{th} }} = a_{1} \times \sin \left( {b_{1} R + c_{1} } \right) $$

where µ_c_ represents the threshold graphene nanoribbons’ chemical potential for sorting and deviating any specific nanoparticles with radii of R. the value of a_1_, b_1,_ and c_1_ are constant and are equal to a_1_ = 43 × 10^–3^ b_1_ = 8 × 10^–3^ and c_1_ = 1.574.

## Conclusion

This article presents a structure for manipulating and sorting nanoparticles using the optical and electrical advantages of graphene nanoribbons embedded in the hBN substrate. In this structure, two methods are used to separate the particles, one is through the distance of the ribbon with the main channel in x direction (vertical to the main channel), and the other is through the chemical potential of graphene. It was also shown that the presented structure could sort particles with a radius below 2.5 nm, making it one of the few structures that can trap and sort nanoparticle particles below 5 nm in diameter. In addition, both methods showed that the presented structure could sort the particles entering the liquid according to their refractive index. The primary physical mechanism of both methods in the proposed structure is to control the force on the particle moving inside the channel, which can be done through the gap between the ribbon and the channel or the potential chemical level of the graphene. The presented structure can be one of the most used structures and open a door for the future of nano-biosensors. Also, this structure makes it possible to sort and manipulate all kinds of viruses or any desired particles in the liquid with very high accuracy of 1 nm. Another advantage of the proposed design is its facility to fabricate, and there is no difficulty in the fabrication process of the proposed structure, or second-order effects and irregularities arise from errors in the fabrication process of the structure. furthermore, by using several animations in the main text and as supplemental information the performance and physical mechanisms of the understanding of the proposed structure became very clear. (Please see the supplementary material for more detail).

## Supplementary Information

Supplementary Information 1.

Supplementary Information 2.

Supplementary Information 3.

Supplementary Video 1.

Supplementary Information 4.

Supplementary Information 5.

Supplementary Information 6.

Supplementary Information 7.

Supplementary Information 8.

Supplementary Information 9.

Supplementary Video 2.

## Data Availability

The datasets used and analyzed during the current study are available from the corresponding author upon reasonable request.
