# Author Correction: Graphene-based optofluidic tweezers for refractive-index and size-based nanoparticle sorting, manipulation, and detection

**DOI:** 10.1038/s41598-023-30740-7

**Published:** 2023-03-14

**Authors:** Elnaz Gholizadeh, Behnam Jafari, Saeed Golmohammadi

**Affiliations:** Faculty of Electrical and Computer Engineering, University of Tabriz, Tabriz, 5166616471 Iran

Correction to: *Scientific Reports* 10.1038/s41598-023-29122-w, published online 03 February 2023

The original version of this Article contained an error in Reference 43,

43. Gholizadeh, E., Jafari, B., Golmohammadi, S. & Soofi, H. in 2022 4th West Asian Symposium on Optical and Millimeter-wave Wireless Communications (WASOWC). 1–6 (IEEE).

now reads:

43. Gholizadeh E., Jafari B., Golmohammadi S., & Soofi H., Low insertion loss and high modulation depth Tunable modulator at Telecommunications Band enabled by graphene/hBN multilayer gratings, in *2022 4th West Asian Symposium on Optical and Millimeter-wave Wireless Communications (WASOWC)*, 1–6. 10.1109/WASOWC54657.2022.9798421 (IEEE, 2022).

The original Article has been corrected.

