# Supplementary figures and images for: Graphene-based optofluidic tweezers for refractive-index and size-based nanoparticle sorting, manipulation, and detection
Source: Sci Rep. 2023 Feb 3;13:1975. doi: 10.1038/s41598-023-29122-w (PMC9898258; doi:10.1038/s41598-023-29122-w)

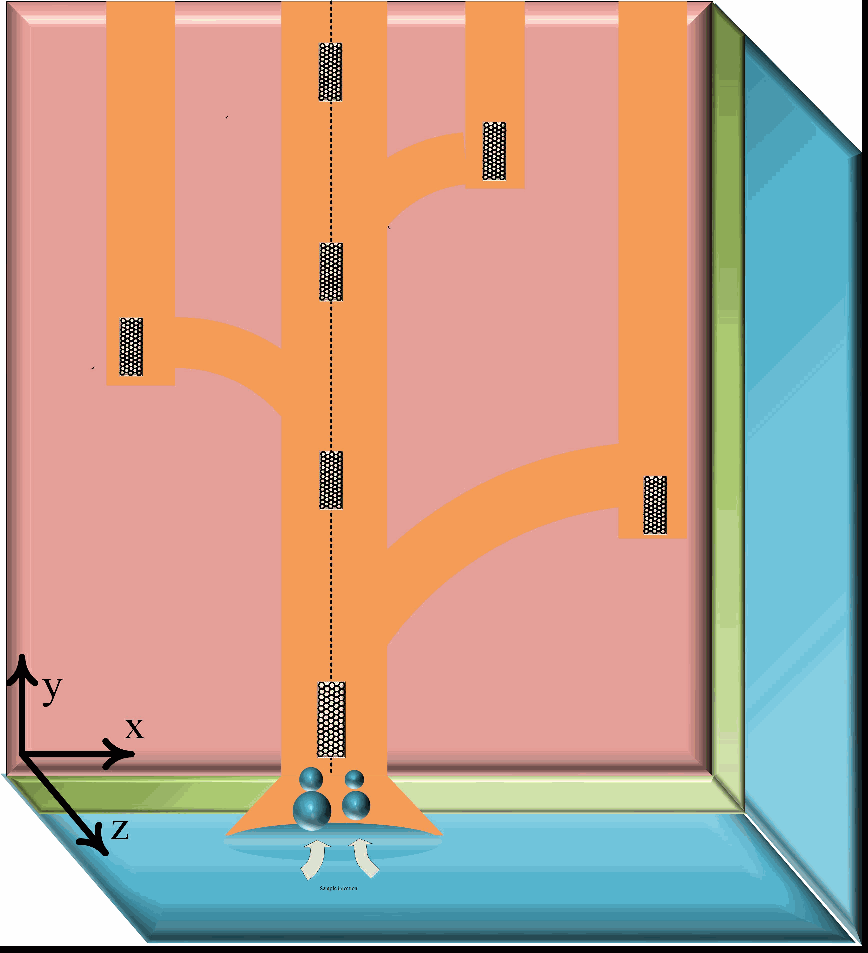

Supplement: Supplementary file 1 — Supplementary Information 1. [file 41598_2023_29122_MOESM1_ESM.gif]

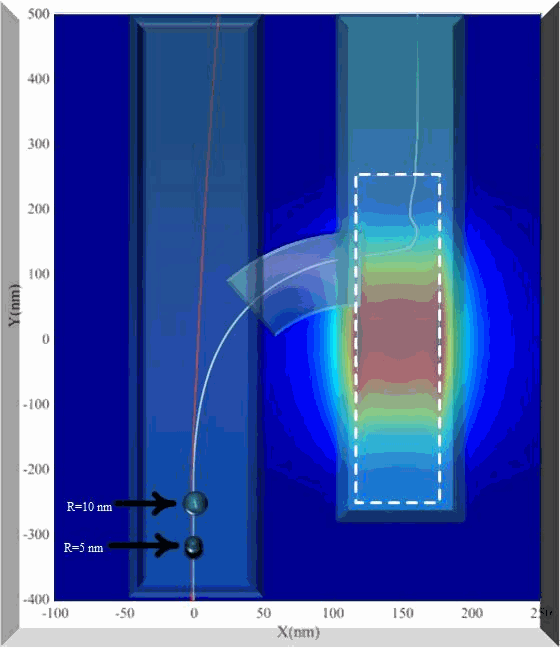

Supplement: Supplementary file 2 — Supplementary Information 2. [file 41598_2023_29122_MOESM2_ESM.gif]

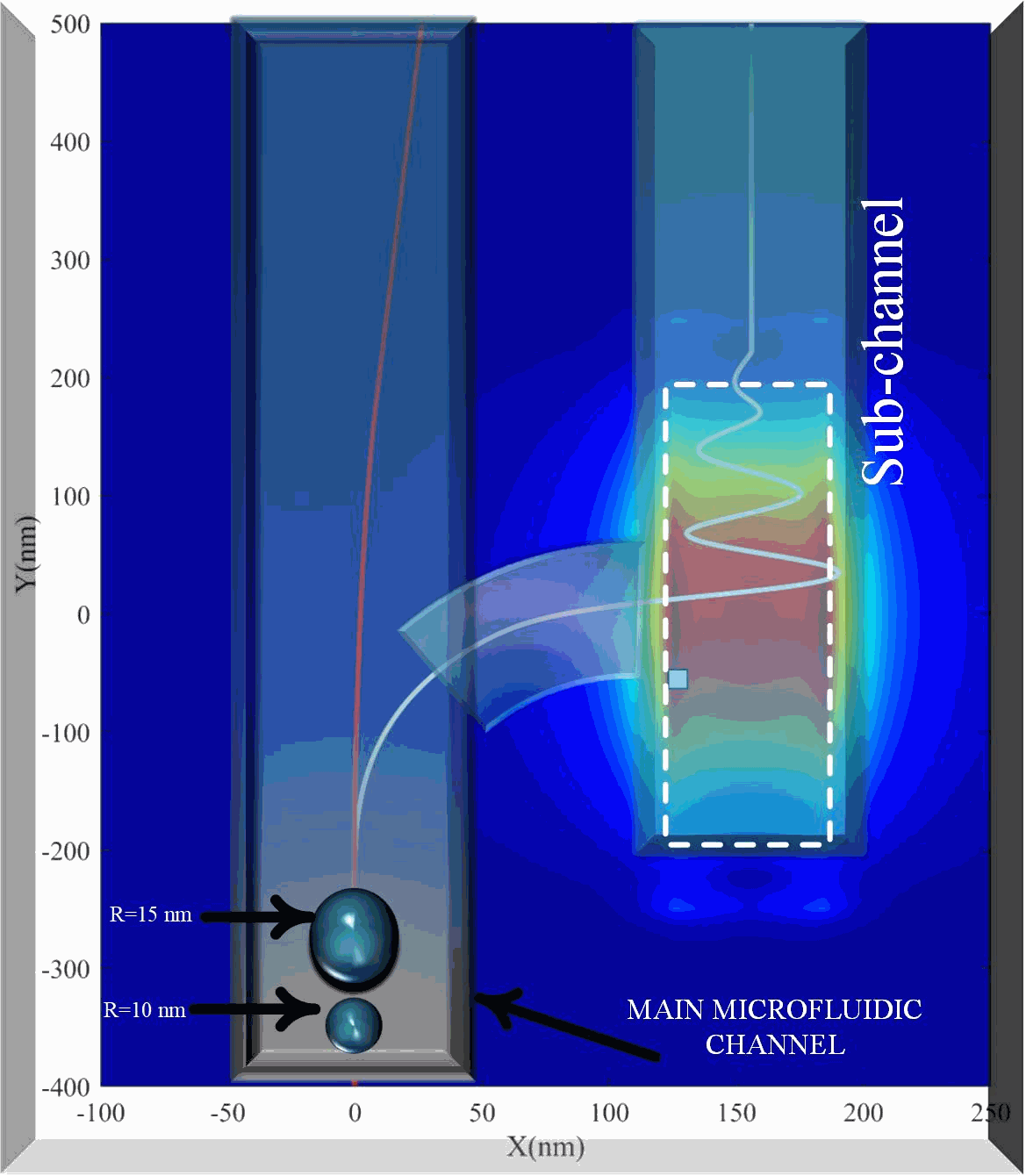

Supplement: Supplementary file 3 — Supplementary Information 3. [file 41598_2023_29122_MOESM3_ESM.gif]

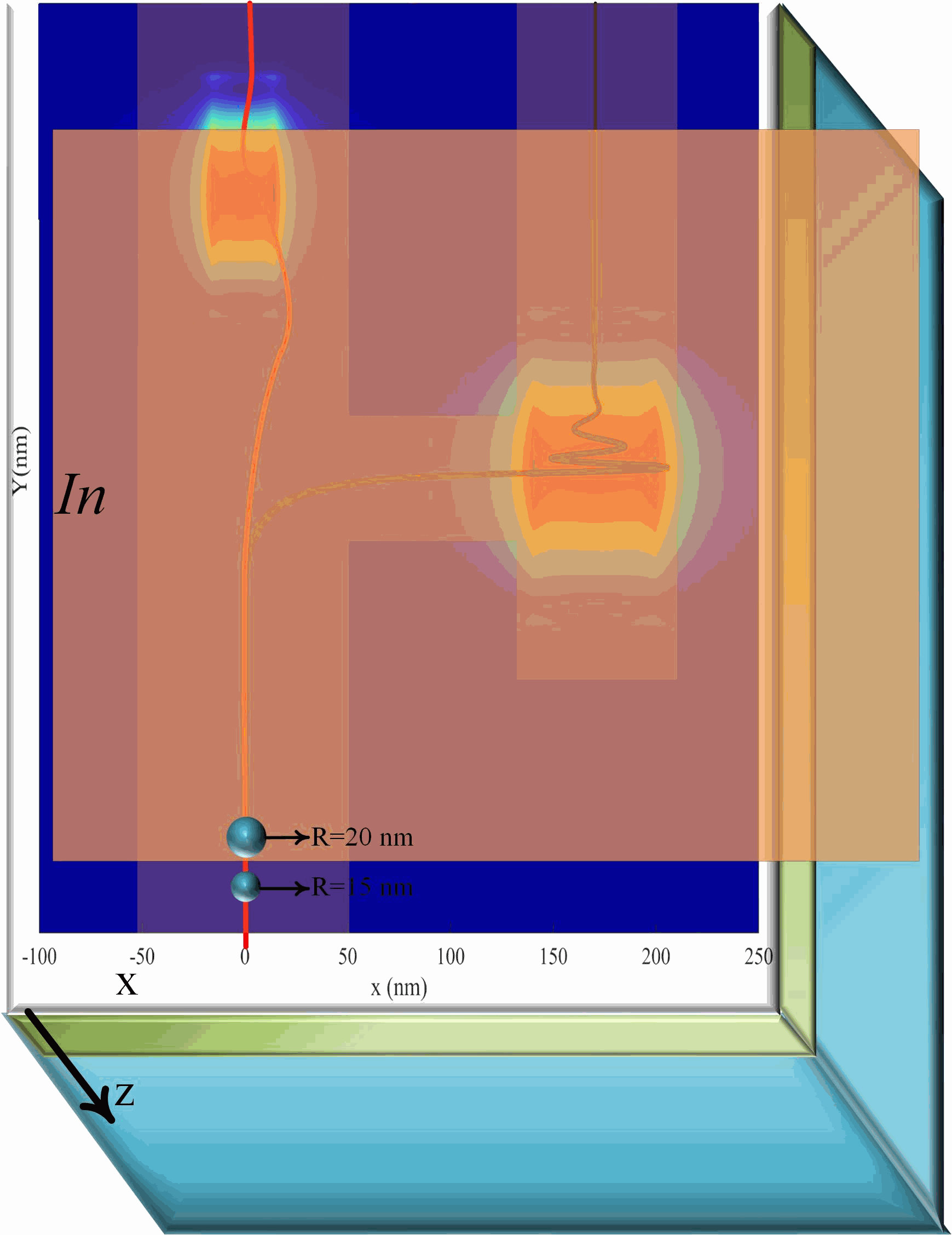

Supplement: Supplementary file 5 — Supplementary Information 4. [file 41598_2023_29122_MOESM5_ESM.gif]

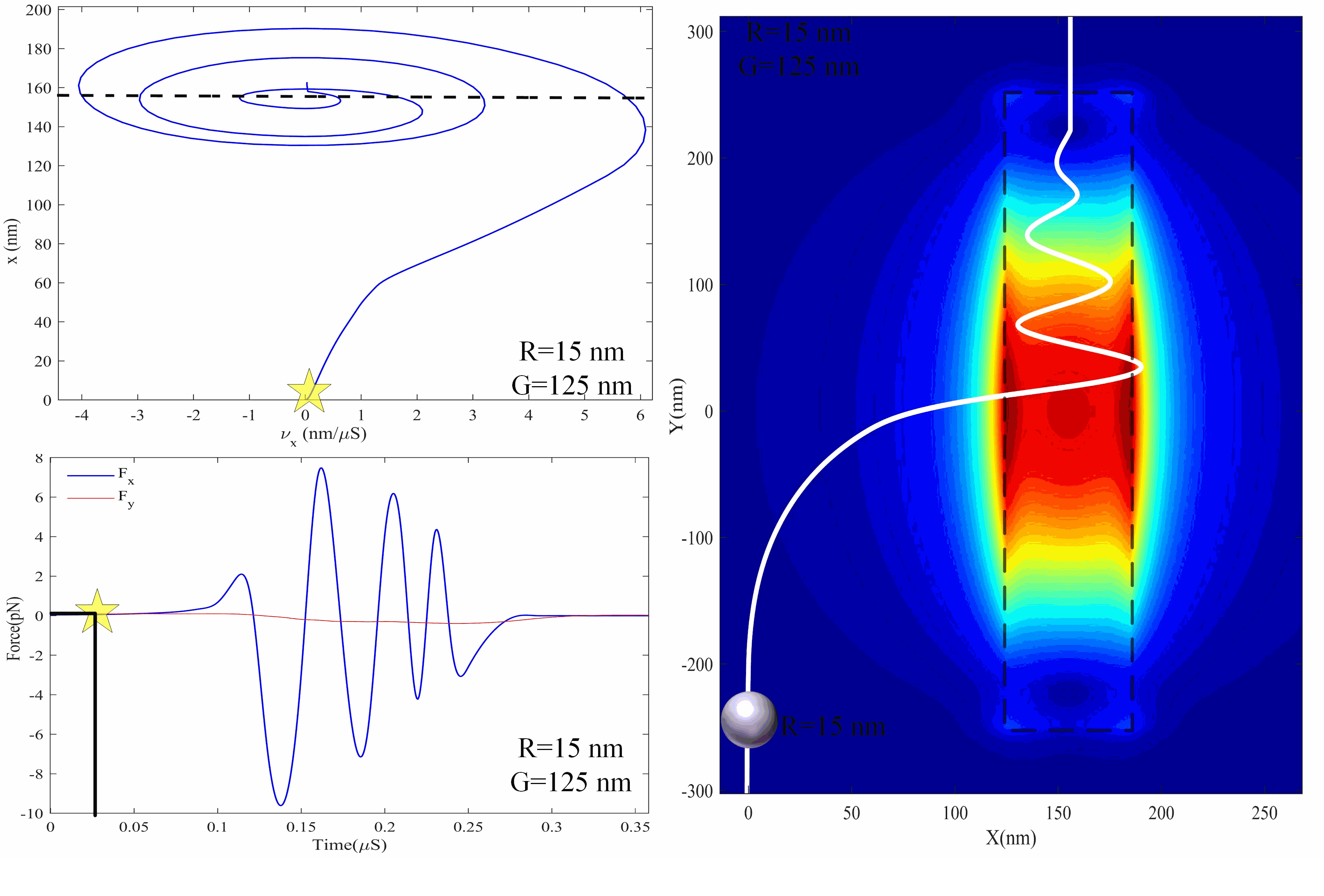

Supplement: Supplementary file 7 — Supplementary Information 6. [file 41598_2023_29122_MOESM7_ESM.gif]

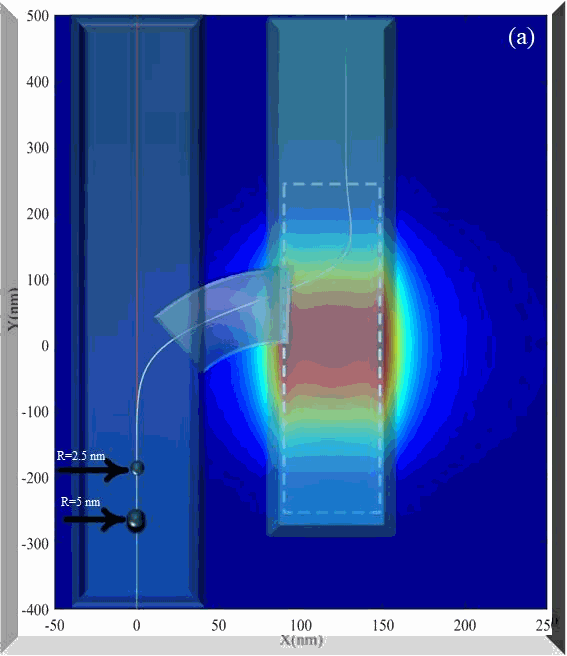

Supplement: Supplementary file 8 — Supplementary Information 7. [file 41598_2023_29122_MOESM8_ESM.gif]

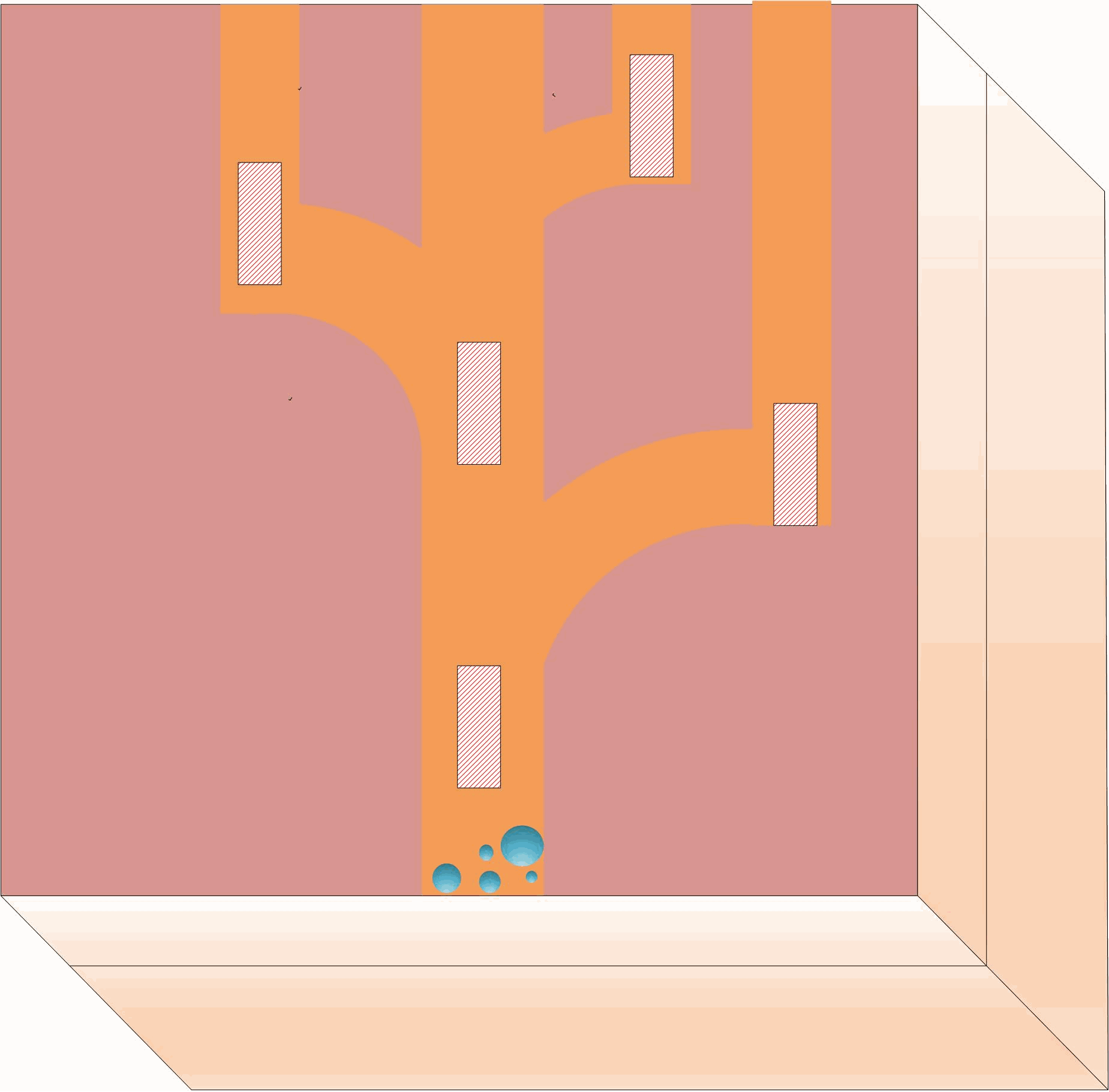

Supplement: Supplementary file 9 — Supplementary Information 8. [file 41598_2023_29122_MOESM9_ESM.gif]
