# Supplementary material for: Graphene-based optofluidic tweezers for refractive-index and size-based nanoparticle sorting, manipulation, and detection
Source: Sci Rep. 2023 Feb 3;13:1975. doi: 10.1038/s41598-023-29122-w (PMC9898258; doi:10.1038/s41598-023-29122-w)
Supplement: Supplementary file 10 — Supplementary Information 9. [file 41598_2023_29122_MOESM10_ESM.docx]

Graphene-based optofluidic tweezers for refractive-index and size-based nanoparticle sorting, manipulation, and detection

Elnaz Gholizadeh ^1, +^, Behnam Jafari ^1*, +^, Saeed Golmohammadi ^1^

^1^ Faculty of Electrical and Computer Engineering, University of Tabriz, Tabriz 5166616471, Iran

*Corresponding author: [behnam.jafari95@ms.tabrizu.ac.ir](mailto:behnam.jafari95@ms.tabrizu.ac.ir)

^+^ **These authors contributed equally to this work**

In the main manuscript, the sorting algorithm of nanoparticles was explained with detailed by the proposed structure. But it may seem complicated. To reduce the complexity and make it easier to understand the physical mechanism of the proposed structure and the way of sorting nanoparticles from an injection liquid to below 2.5 nm, an animation has been designed in which all the steps explained in the main text are shown in animation form.

Gif. 1 represent the explained mechanism for sorting nanoparticles. As can be seen at first nanoparticles are pushed into a microfluidic channel. The first graphene nanoribbon causes that, when microfluidic is injected into the channel, all the nanoparticles accumulate in the center of the main channel, which is shown by the dashed dot in Fig. 1 (c). therefore, since there is not any exerted optical force arising by the graphene nanoribbon in the y direction, the exerted optical force in the x direction pushes the nanoparticles to the center of the graphene nanoribbon, and all the nanoparticles are trapped in the x direction but continues to move in the Y direction in respect to microfluidic force.

When the first larger size particle is sorted the smaller ones also deviate from their path, therefore it is necessary to put graphene nanoribbons after each step or sorting to push back the particles to the center of the graphene nanoribbon or main channel. Also, as you can see, the smaller particles are ahead of the larger particles, the reason is that the smaller nanoparticles move with larger velocity due to higher acceleration. In the following, the physical mechanisms of nanoparticle sorting in two steady state and dynamic response states are described in detail. (Please see the supplementary information for more details)


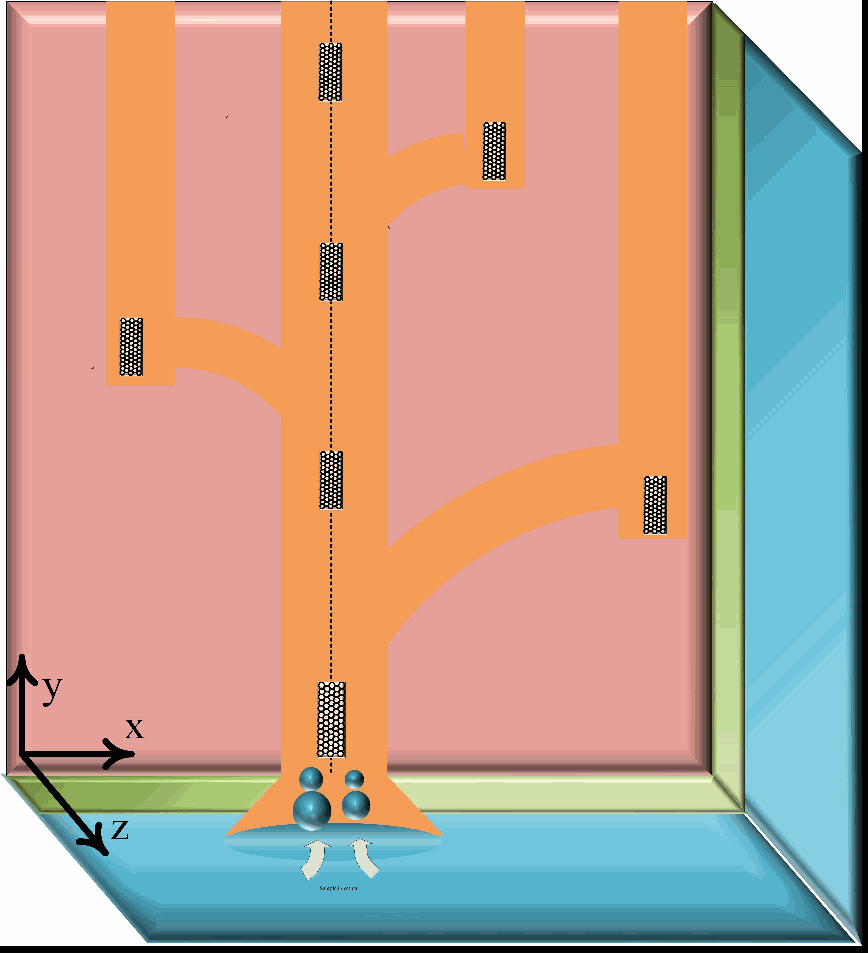


*Gif. 1 the physical mechanism and sorting algorithm of nanoparticles using the proposed novel method.*

To further open up the application of the proposed structure and how the nanoparticles are trapped in the subchannels in the x direction, here we have another animation for a nanoparticle with a radius of 15 nm along with displacement of R=10 nm nanoparticle as shown in Fig.13 (c) of the main manuscript. As can be seen from gif. 2, the presented structure is based on a realistic and real platform, and despite all its complications, it can build and open a new door on optical tweezers.

The force sign in the x direction is also shown in Gif. 2. As can be seen R=15 nm nano particle’s F_x_ has been increasing until it reaches the edge of the graphene nanoribbon and it experienced the maximum value of F_x_ (x=125 nm), which exactly matches Fig. 7 (b) of the main manuscript, after passing the edge of the nanoribbon when the particle moves on the graphene nanoribbon its positive F_x_ sharply reduces to zero in the center of graphene nanoribbon (x=155 nm). after passing the center of the graphene nanoribbon the negative force is exerted on the nanoparticle, but the particle moves toward the +x direction due to its momentum. The particle velocity decreases due to negative F_x_ until its velocity reaches zero at almost the other side of the graphene nanoribbon. But again, the nanoparticle due to +F_x_ pushed back toward the center of the graphene nanoribbon, while its F_x_ reaches zero at the center of the ribbon but due to its initial momentum it passes the center of the nanoribbon and felt again +F_x_ but with lower velocity. These steps are repeated a few times until the particle’s velocity became zero exactly on the center of the graphene nanoribbon and it reaches a steady state where there is no velocity or force in the x direction, but the nanoparticles are free in the y direction and the drag force pushes the particle toward the outlet.

On the other hand, the smaller nanoparticle with a radius of 10 nm, which is not the target of sorting, is also affected by the vertical force caused by the graphene nanoribbon (in the +x direction). But as can be seen from Gif. 2 and Figure 13. (c) of the main manuscript, the amount of deviation is too small, in comparison with the target nanoparticles (in this example as mentioned above paragraph is R=15 nm nanoparticles) but this small amount also causes the inefficiency of the proposed structure in long distances. Therefore, the solution to this problem is to place nanoribbons in the center of the graphene after each sorting of the target particles, which can remove the unwanted deviations in the smaller particles and collect all remained unsorted nanoparticles, again in the center of the main channel. As shown in Gif. 2, a 15 nm radius nanoparticle perfectly deviated and trapped in the x direction on subchannel graphene nanoribbon then keep moving in the y direction concerning liquid force. But the smaller nanoparticles experienced an undesired deviation. As mentioned, this small undesired deviation will be solved at the next step using a graphene nanoribbon between each step. These steps are repeated until only nanoparticles with a radius smaller than 2.5 nm remain in the main channel. Therefore, it can be said that the proposed structure is capable of sorting nanoparticles with a radius of less than 2.5 nm nanoparticles.


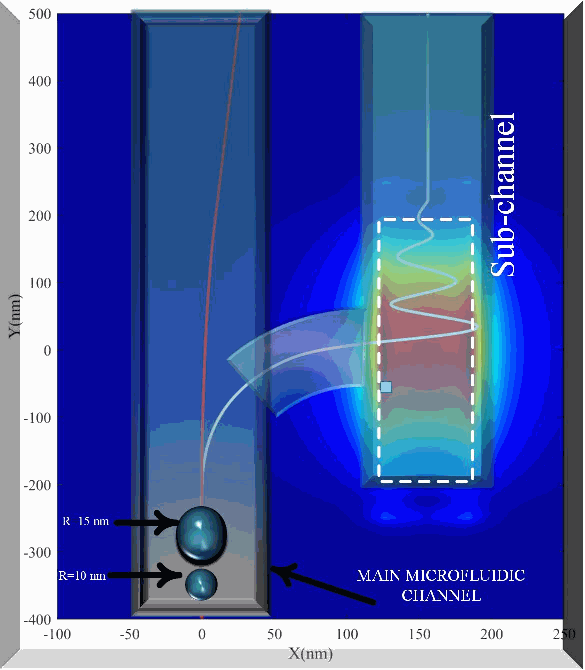


*Gif. 2 An animation of sorting R=15 nm nanoparticles and how it reaches steady states along with the undesired deviation of R=10 nm nanoparticles.*

Below in Gif. 3 the dynamic response of R=20 and 15 nm has been provided with more detail. As shown in Fig .13 (d) of the main manuscript, Although R=20 nm has been sorted from the main channel by fixing the G_th_ to 140 nm, on the other side the smaller particle which has a radius of R=15 nm owns an undesired deviation of Δx=25 nm, and as can be seen, it reaches to the edge of the main channel, which is not desirable. Therefore, the graphene nanoribbons located in the main channel functionality can be seen clearly here which is how it prevents moving R=15 nm nanoparticles in the unwanted devotion path.

In this animation, the steps and method of sorting nanoparticles with a radius of 20 nm are shown. Also, the dynamic response of 20 nm and 15 nm nanoparticles is shown (according to Fig.13 (d)). The use of graphene nanoribbons placed in the center of the main channel can be clearly seen in this animation.


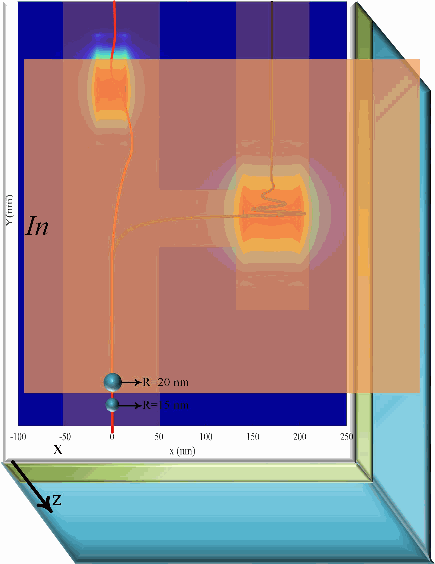


*Gif.* *3 the dynamic response of R=15 and 20 nm as Fig.13 (d) with graphene nanoribbon in the main channel and its effect to the pushing back undesired deviations.*

As it is shown in Fig.14 (c) of the main manuscript the velocity of nanoparticles has a circular form, the reason is the sinusoidal pattern of force for a long time and the nanoparticle position when it is trapping by nanoribbon in the x-direction at the subchannel. Below Gif.4 represent the relations between the dynamic response of a nanoparticle with R=15 nm and its position with its velocity along time and the force exerted on the nanoparticle as a function of time. This Gif links all these important elements to sorting a nanoparticle and gives a better illustration and understanding of how the proposed method work.


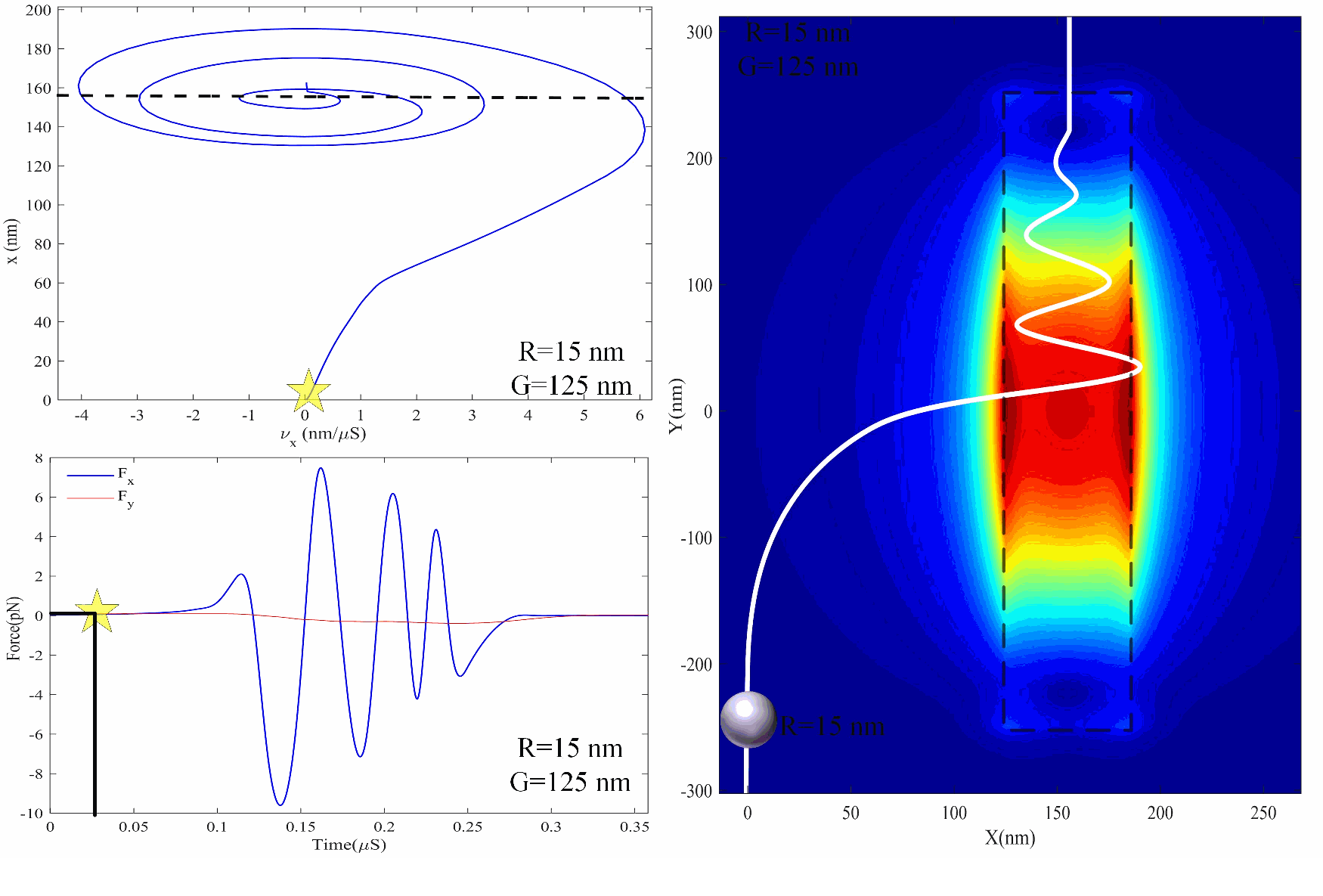


*Gif. 4 the dynamic response of a nanoparticle with R=15 nm and its position-based velocity along with the force felt by the nanoparticle as a function of time.*

In Gif. 1 it can be seen that the subchannels are not separated from the main channel. All of the channels are connected to microfluidic channels. In another word the sample injected into the main channel with a liquid, paths the subchannel too, therefore the deviated nanoparticles due to induced force in x direction (vertical force) easily move with microfluidic. Below Gif. 5 represents The dynamic response of R=2.5 and 5 nm (Fig.13 (a) of the main manuscript)


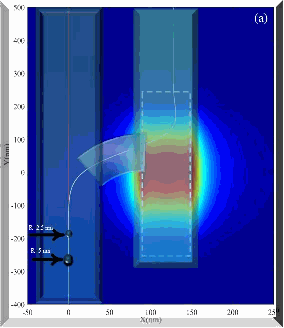


*Gif. 5 A 2D animation of sorting R=5 nm nanoparticle and the undesired deviation of R=2.5 nm nanoparticle according to Fig.13. (a) of the main text.*

From the above Gifs, it can be understood that as particles became smaller the sinusoidal patterns disappear slowly. Below the dynamic response of R=5 nm and R=10 nm nanoparticles in which the 10 nm nanoparticle has been sorted is shown.


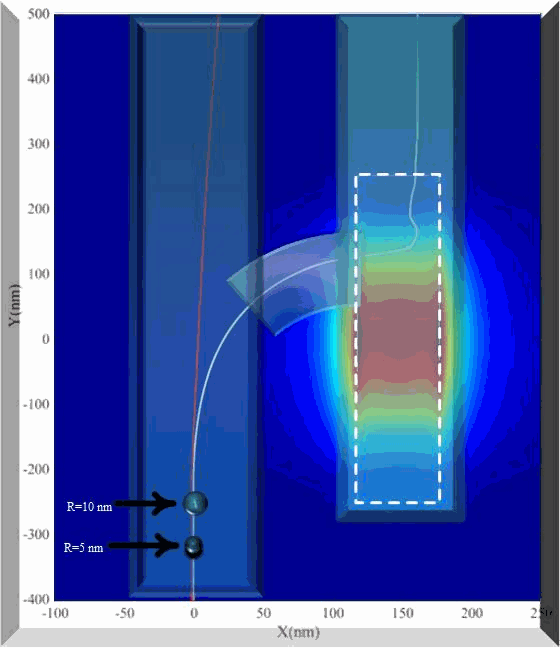


*Gif. 6 2D animation of dynamic response of R=10 and 5 nm nanoparticles in a fixed gap according to Fig.13 (b) of the main text in which R=10 nm nanoparticle due to vertical force of graphene nanoribbon located in the subchannel in the +x direction is deviated from main channel to subchannel and filtered out (sorted) from other nanoparticles.*
